# STAMP2 increases oxidative stress and is critical for prostate cancer

**DOI:** 10.15252/emmm.201404181

**Published:** 2015-02-13

**Authors:** Yang Jin, Ling Wang, Su Qu, Xia Sheng, Alexandr Kristian, Gunhild M Mælandsmo, Nora Pällmann, Erkan Yuca, Ibrahim Tekedereli, Kivanc Gorgulu, Neslihan Alpay, Anil Sood, Gabriel Lopez-Berestein, Ladan Fazli, Paul Rennie, Bjørn Risberg, Håkon Wæhre, Håvard E Danielsen, Bulent Ozpolat, Fahri Saatcioglu

**Affiliations:** 1 Department of Biosciences, University of OsloOslo, Norway; 2 Institute for Cancer Genetics and Informatics, Oslo University HospitalOslo, Norway; 3 Department of Tumor Biology, Oslo University HospitalOslo, Norway; 4 Department of Experimental Therapeutics, MD Anderson Cancer CenterHouston, TX, USA; 5 Gynecological Oncology, MD Anderson Cancer CenterHouston, TX, USA; 6 The Vancouver Prostate CentreVancouver, BC, Canada; 7 Division of Pathology, Oslo University HospitalOslo, Norway; 8 Division of Surgery, Oslo University HospitalOslo, Norway; 9 Center for Cancer Biomedicine, University of OsloOslo, Norway; 10 Department of Informatics, University of OsloOslo, Norway

**Keywords:** activating transcription factor 4, iron reductase, prostate cancer, reactive oxygen species, six transmembrane protein of prostate 2

## Abstract

The six transmembrane protein of prostate 2 (*STAMP2)* is an androgen-regulated gene whose mRNA expression is increased in prostate cancer (PCa). Here, we show that STAMP2 protein expression is increased in human PCa compared with benign prostate that is also correlated with tumor grade and treatment response. We also show that STAMP2 significantly increased reactive oxygen species (ROS) in PCa cells through its iron reductase activity which also depleted NADPH levels. Knockdown of STAMP2 expression in PCa cells inhibited proliferation, colony formation, and anchorage-independent growth, and significantly increased apoptosis. Furthermore, STAMP2 effects were, at least in part, mediated by activating transcription factor 4 (ATF4), whose expression is regulated by ROS. Consistent with *in vitro* findings, silencing *STAMP2* significantly inhibited PCa xenograft growth in mice. Finally, therapeutic silencing of *STAMP2* by systemically administered nanoliposomal siRNA profoundly inhibited tumor growth in two established preclinical PCa models in mice. These data suggest that STAMP2 is required for PCa progression and thus may serve as a novel therapeutic target.

## Introduction

Prostate cancer (PCa) is the most frequently diagnosed non-skin cancer and second leading cause of cancer deaths among men in Europe and the USA (Siegel *et al*, 2012). PCa growth is initially dependent on circulating androgens and hormonal therapies aimed at androgen deprivation result in regression. However, in the majority of cases, the disease recurs as a castration-resistant PCa (CRPC) that leads to death regardless of available treatment options. Despite recent advances, the molecular mechanisms involved in PCa development and progression to CRPC are still not well understood (Arnold & Isaacs, 2002; Shen & Abate-Shen, 2010; Dayyani *et al*, 2011; Yap *et al*, 2011). This information is necessary for biomarker discovery for disease stratification as well as identifying therapeutic targets for PCa (Prensner *et al*, 2012).

Previous studies have shown that androgen signaling, mediated by the androgen receptor (AR), has a role in all phases of PCa, including CRPC (Arnold & Isaacs, 2002; Shen & Abate-Shen, 2010; Dayyani *et al*, 2011; Yap *et al*, 2011; Bluemn & Nelson, 2012). AR activation regulates a large cluster of genes involved in multiple aspects of cellular function. Genome-wide explorations have revealed that several hundred genes are primary targets of AR in PCa cells (Velasco *et al*, 2004; Lai *et al*, 2010). In addition, deregulated androgen signaling increases reactive oxygen species (ROS) in PCa (Ripple *et al*, 1997; Sun *et al*, 2001; Tam *et al*, 2003; Frohlich *et al*, 2008; Basu *et al*, 2009), consistent with other work which suggests that PCa development is associated with oxidative stress (for a review, see Paschos *et al*, 2013). However, the precise molecular events that cause changes in the generation of ROS in PCa are currently not known.

We have previously identified *STAMP2* as an androgen-regulated gene (Korkmaz *et al*, 2005). *STAMP2* mRNA is expressed in the prostate epithelium and is significantly overexpressed in PCa compared with benign prostate; consistently, ectopic expression of STAMP2 promoted PCa cell proliferation (Korkmaz *et al*, 2005). Although these observations suggested that STAMP2 may have a role in PCa biology, it is not clear as to how STAMP2 modulates cell growth in PCa cells and whether it can serve as a therapeutic target. Interestingly, STAMP2 has also been linked to inflammation and insulin action in adipocytes and macrophages and thus has been implicated in metabolic disease and atherosclerosis (Wellen *et al*, 2007; ten Freyhaus *et al*, 2012).

Here, we show that STAMP2 is a critical survival factor for PCa cells *in vitro* and *in vivo* and that it activates oxidative stress-induced ATF4 signaling through ROS generated by its iron reductase activity. Consistently, therapeutic silencing of *STAMP2* in two established preclinical PCa models in mice by nanoliposomal siRNA delivery results in profound tumor regression.

## Results

### STAMP2 expression is up-regulated in human PCa specimens

**Figure 1:**
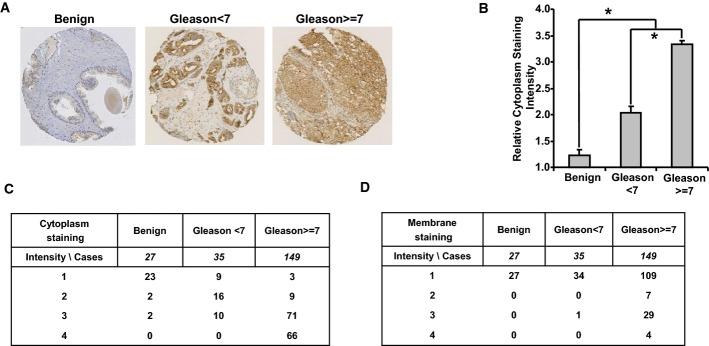
STAMP2 expression during PCa progression
A Immunohistochemistry was used to assess STAMP2 expression in normal and malignant human prostate specimens. Tissue microarrays (TMAs) with normal prostate (*n *=* *27) and early-stage (Gleason grade < 7) (*n *=* *35) or late-stage prostate tumors (Gleason grade ≥ 7) (*n *=* *149) were subjected to immunohistochemistry as described in Materials and Methods. Representative images are shown.B Quantification of cytosolic STAMP2 staining in TMAs is shown in (A). One-way analysis of variance (ANOVA) with a *post hoc* test, *P < 0.0001. Error bars indicate SEM.C, D The details of cytosolic (C) and membrane (D) STAMP2 staining in the TMAs described in (A).

We have previously shown that *STAMP2* mRNA expression is increased in PCa compared with benign prostate (Korkmaz *et al*, 2005). To evaluate the validity of this finding at the protein level, we assessed STAMP2 expression by immunohistochemistry on a tissue microarray (TMA) that contained benign prostate tissue (*n* = 27) and malignant prostate tissue with Gleason score < 7 (*n* = 35) or ≥ 7 (*n* = 149). Benign prostate tissue showed low STAMP2 staining, while STAMP2 expression was significantly increased in PCa compared to normal cells (Fig1A). In addition, STAMP2 cytosolic staining correlated with the tumor grade (Gleason score) (Fig1B). Of interest, STAMP2 staining was restricted to the cytosol in low-grade prostate tumors, whereas its cell membrane localization significantly increased in high-grade tumors (Fig1C and D).

### STAMP2 has a critical role in PCa growth both *in vitro* and *in vivo*

**Figure 2:**
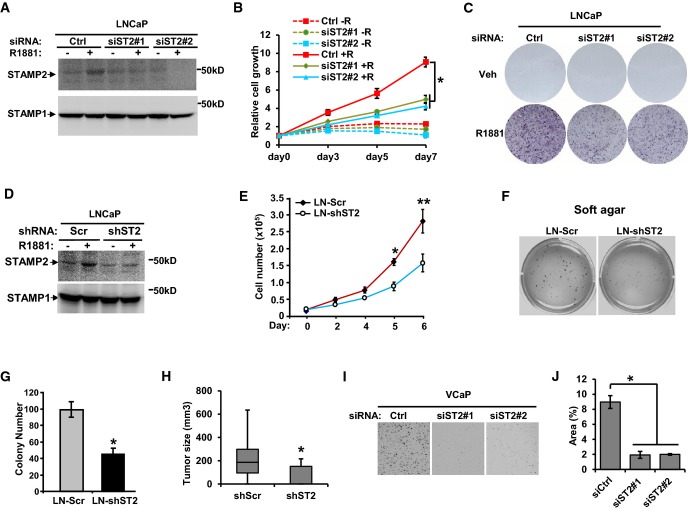
STAMP2 promotes PCa growth *in vitro* and *in vivo*
A LNCaP cells were transfected with either control siRNA or two independent STAMP2 siRNAs, and membrane fractions of the cells were prepared followed by Western blotting analysis. STAMP1 is used as a loading control.B LNCaP cells were cultured in RPMI 1640 medium containing 10% CT-FBS and treated with or without 1 nM R1881 for 24 h before being transfected with the indicated siRNAs. The cells were then cultured for the indicated times, and cell growth was measured by Cell Counting Kit-8. *n* = 3, *P < 0.0001. Error bars indicate SD.C LNCaP cells were cultured and transfected with the indicated siRNAs as described in (B). After transfection, the cells were cultured for 10 days. The colonies formed were stained and photographed.D LNCaP cells stably expressing shRNA against STAMP2 or control shRNA were treated with or without 1 nM R1881. The cells were then collected, and membrane fractions were prepared followed by Western blot analysis.E LNCaP cells stably expressing shRNA against STAMP2 or control shRNA were cultured for the indicated times, and the cell numbers were determined using a hemocytometer. *n* = 3, *P =* *0.047; **P =* *0.0046. Error bars indicate SD.F LNCaP cells stably expressing shRNA against STAMP2 or control shRNA were cultured in soft agar for 2 weeks as described in Materials and Methods. The plates were then stained and photographed.G Quantification of data from (F). *n *=* *3, *P =* *0.022. Error bars indicate SD.H LNCaP cells stably expressing shRNA against STAMP2 or control shRNA were subcutaneously implanted into male SCID mice. Tumor size was measured after 8 weeks. *n *=* *9, *P =* *0.037. Error bars indicate SEM.I VCaP cells were transfected with the indicated siRNA as described in (B). After transfection, VCaP cells were cultured for 2 weeks. The colonies formed were stained and photographed.J Quantification of the data from (I). *n* = 3, *P < 0.0001. Error bars indicate SD.
Data information: In (B, E, G, H, and J), Student's *t*-test was performed to analyze the statistical significance. Source data are available online for this figure.

We have previously found through ectopic expression in DU145 PCa cells that STAMP2 may have a role in cell proliferation (Korkmaz *et al*, 2005). To evaluate this further, we assessed whether reduction in endogenous STAMP2 expression influences growth characteristics of PCa cells. STAMP2-specific siRNAs significantly decreased STAMP2 expression compared with control siRNA in LNCaP cells (Fig2A and Supplementary Fig S1A). Knockdown of STAMP2 significantly inhibited androgen-dependent growth of LNCaP cells measured by cell number and colony formation capacity (Fig2B and C). Consistently, LNCaP cells stably expressing STAMP2 shRNA displayed reduced growth rates both *in vitro* (Fig2D–G) and *in vivo* (Fig2H).

To generalize these observations, we validated the contribution of STAMP2 to cell growth in an additional androgen-responsive PCa cell line, VCaP. Similarly, in LNCaP cells, knockdown of STAMP2 (Supplementary Fig S1B) significantly inhibited VCaP cell growth (Fig2I and J). Collectively, these data show that STAMP2 has an important role in PCa cell growth *in vitro* and *in vivo*.

### STAMP2 affects cell cycle progression of PCa cells and their sensitivity to apoptosis-inducing agents

**Figure 3:**
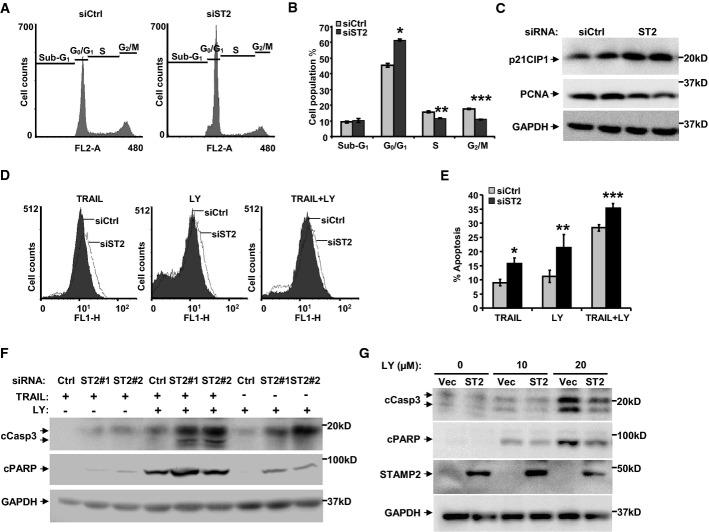
STAMP2 affects cell cycle progression and increases sensitivity of PCa cells to apoptosis-inducing drugs
A LNCaP cells were transfected with either control or STAMP2-specific siRNA. Two days after transfection, cells were subjected to cell cycle analysis as described in Materials and Methods. Representative histograms showing PI-stained cells are shown.B The proportion of cells in each stage of the cell cycle from the experiment in (A) is presented. Student's *t*-test was performed to analyze the statistical significance, *n *=* *3. *P =* *0.0002; **P =* *0.0056; ***P =* *0.0099. Error bars indicate SD.C Two days after transfection with the indicated siRNAs, LNCaP cells were harvested and cell lysates were prepared and subjected to Western blot analysis with the indicated antisera. Representative blots of three independent experiments are shown.D LNCaP cells were transfected with either control or STAMP2-specific siRNA. Three days after transfection, cells were treated with either 50 ng/ml TRAIL or 20 μmol/l LY294002 (LY) for 24 h, or both agents for 6 h, and then subjected to TUNEL/FACS analysis. Representative histograms of TUNEL-stained cells are shown. FL1-H refers to the gating of the cells for the TUNEL staining with fluorescence measurement.E The extent of apoptosis from the experiment in (D) is presented. Student's *t*-test was performed to analyze the statistical significance, *n *=* *3. *P =* *0.027; **P =* *0.0058; ***P =* *0.0016. Error bars indicate SD.F LNCaP cells were transfected with either control or two independent STAMP2-specific siRNAs, ST2-1 and ST2-2. Three days after transfection, cells were treated in the same way as in (D). Whole-cell lysates were prepared and subjected to Western blot analysis with the indicated antisera.G LNCaP cells stably expressing either control or STAMP2 vector were treated in the same way as in (D). Cell lysates were prepared and subjected to Western blot analysis with the indicated antibodies.
Source data are available online for this figure.

We next evaluated whether the growth stimulatory effects of STAMP2 could be mediated by impacting the cell cycle. As shown in Fig3A and B, upon STAMP2 knockdown, there was a significant increase in the percentage of cells in G0/G1, indicating that loss of STAMP2 led to partial cell cycle arrest. Consistently, expression of CDK inhibitor p21CIP1 was up-regulated, while the proliferation marker proliferating cell nuclear antigen (PCNA) was down-regulated by STAMP2 knockdown (Fig3C).

In addition to its effects on proliferation, we investigated the possible involvement of STAMP2 in regulating apoptosis in PCa cells. No significant changes in basal levels of apoptosis were detected upon STAMP2 knockdown (data not shown). We then determined whether STAMP2 knockdown influences the sensitivity of LNCaP cells to apoptosis inducers, tumor necrosis factor (TNF)-related apoptosis-inducing ligand (TRAIL) (Yu *et al*, 2000) and PI3K inhibitor (LY294002) (Chen *et al*, 2001). LNCaP cells were transfected with either control siRNA or two independent siRNAs against STAMP2, and the cells were then treated with either TRAIL or LY294002 alone, or in combination, and the extent of apoptosis was determined. STAMP2 knockdown significantly increased cell death induced by TRAIL or LY294002 alone, as well as TRAIL plus LY294002 (Fig3D and E). Consistent with a role in regulating apoptosis, STAMP2 knockdown increased cleavage of PARP and caspase-3 in response to TRAIL, LY294002, or their combination (Fig3F, Supplementary Fig S1C and D). Conversely, ectopic expression of STAMP2 attenuated caspase-3 and PARP cleavage induced by LY294002 compared to control (Fig3G). These data show that STAMP2 inhibits pro-apoptotic factor signaling in PCa cells and thereby contributes to cell survival.

### STAMP2 is associated with the development of hormone refractory PCa

**Figure 4:**
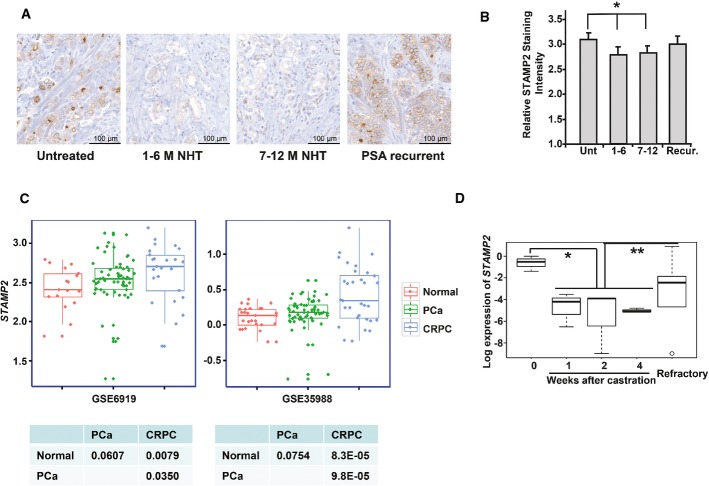
STAMP2 expression is associated with development of castration resistance of PCa
A STAMP2 expression was determined by IHC of a neoadjuvant hormone therapy (NHT) TMA containing samples from hormone naïve (untreated) (*n *=* *28), NHT 1–6 months (*n *=* *31), NHT 7–12 months (*n *=* *41), and patients with PSA recurrence (*n *=* *21) as indicated. Representative images are presented.B Quantitative presentation of the data from (A). Unt, untreated; Recur, PSA recurrence. The Kruskal–Wallis test was used for significance analysis. *P =* *0.0499. Error bars indicate SEM.CBoxplots of *STAMP2* expression levels in normal prostate tissue, primary prostate cancer, and metastatic CRPC were analyzed in two independent GEO datasets (GSE6919 and GSE35988). The values of individual samples are presented as dots. Thick horizontal lines represent the median, with the box representing the upper and lower quartile. The whiskers represent the 5^th^ and 95^th^ percentiles. The statistical significance, indicated in the tables at the bottom, was determined by Student's *t*-test.D CWR22 xenografts were grown in nude mice, and tumors were collected at different times after castration. mRNA was extracted from the tumors and used for qPCR analysis of *STAMP2* expression. The results are presented as boxplots. Thick horizontal lines represent the median, with the box representing the upper and lower quartile. The whiskers represent the 5^th^ and 95^th^ percentiles, and the outlier is presented as an open circle. The statistical significance was determined by one-way ANOVA with a *post hoc* test. *n *=* *3 in group week 0, 1, 2, and 4; *n *=* *5 in refractory group. *P =* *0.011; **P =* *0.049. Error bars indicate SEM.

Because *STAMP2* is an androgen-regulated gene in PCa cells (Korkmaz *et al*, 2005) and contributes to their androgen-dependent growth (Fig2), we set out to examine STAMP2 expression in PCa patients treated with neoadjuvant hormone therapy. STAMP2 levels were significantly reduced following neoadjuvant hormone therapy and remained low in patients responding to therapy (Fig4A and B). Interestingly, upon PSA recurrence, STAMP2 levels were again increased (Fig4A and B). As PSA recurrence is associated with the development of CRPC, we next examined *STAMP2* expression in PCa cohorts that included matched primary PCa and CRPC tissues. As shown in Fig4C, in two independent cohorts, there was an increase in *STAMP2* expression in primary PCa tissues, compared to normal prostate. Furthermore, *STAMP2* expression was significantly higher in CRPC compared to primary PCa. Consistently, *STAMP2* expression was significantly reduced shortly after castration in human PCa xenograft CWR22 grown in immunodeficient mice and then increased in the refractory derivatives (Fig4D). Collectively, these data suggest that STAMP2 expression is associated with CRPC development.

### STAMP2 is involved in androgen-insensitive PCa growth

**Figure 5:**
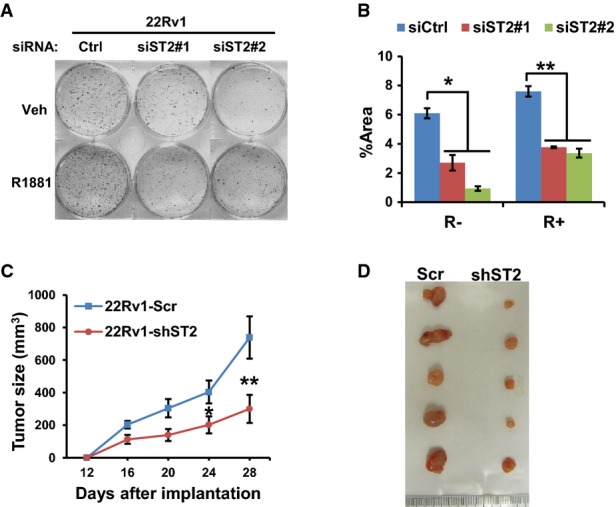
STAMP2 loss inhibits androgen-insensitive PCa cell growth *in vitro* and *in vivo*
A 22Rv1 cells were cultured in RPMI 1640 medium containing 10% CT-FBS and treated with or without 1 nM R1881 for 24 h and were then transfected with the indicated siRNAs. The cells were then cultured for 10 days. The colonies formed were stained and photographed.B Quantification of data from (A). Student's *t*-test was performed to analyze the statistical significance, *n *=* *3. *P =* *0.0003; **P < 0.0001. Error bars indicate SD.C 22Rv1 cells from Supplementary Fig S2 were subcutaneously implanted into both flanks of male nude mice (six mice per group). Tumor size was measured at the indicated time points. Student's *t*-test was performed to analyze the statistical significance, *n *=* *10. *P =* *0.037; **P =* *0.039. Error bars indicate SEM.D Representative tumors from (C) right after resection at the final time point are shown.

As STAMP2 expression is significantly increased in CRPC, we next assessed the possible function of STAMP2 in 22Rv1 cells, a CRPC model cell line derived from the refractory CWR22R xenografts that grow in an androgen-independent manner *in vitro* and *in vivo* (Sramkoski *et al*, 1999). STAMP2 knockdown significantly reduced 22Rv1 growth in the presence or absence of androgen (Fig5A and B). To assess the validity of these findings *in vivo*, we generated cell lines that stably express either scrambled shRNA or shRNA targeting STAMP2. Consistent with the data obtained with siRNA, STAMP2 shRNA expressing cells grew significantly less colonies compared with cells expressing scrambled shRNA (Supplementary Fig S2A and B). Furthermore, when grown as xenografts in nude mice, STAMP2 knockdown cells developed significantly smaller tumors compared with control cells (Fig5C and D). These data are consistent with those from above and suggest that STAMP2 is associated with CRPC.

### STAMP2 is necessary for ATF4 signaling

**Figure 6:**
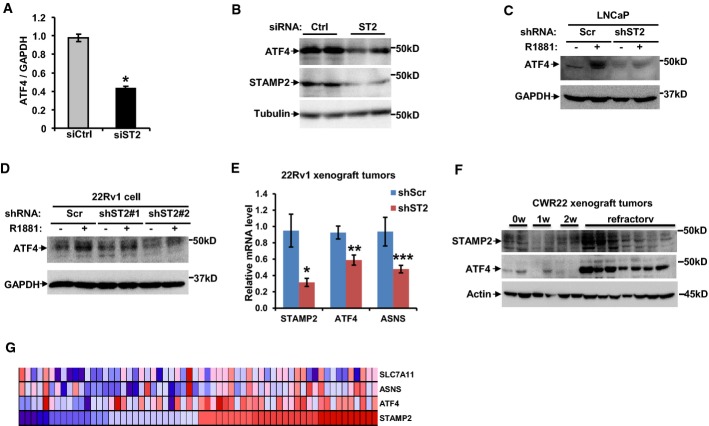
STAMP2 affects ATF4 expression in PCa cells
A LNCaP cells were transfected with either control or STAMP2-specific siRNA in the presence of 10^−8^ M R1881. RNA was isolated, and qPCR was used to determine *ATF4*mRNA levels. Student's *t*-test was used to analyze the statistical significance, *n *=* *3. *P =* *0.002. Error bars indicate SD.B Cell lysates from (A) were subjected to Western blot analysis.C, D ATF4 expression in LNCaP (C) and 22Rv1 (D) cells stably expressing control shRNA or shRNA against STAMP2 was analyzed by Western blot analysis.E *ATF4* expression in xenografted tumors of 22Rv1 cells stably expressing control shRNA or shRNA against STAMP2 was analyzed by qPCR. Student's *t*-test was used to analyze the statistical significance, *n *=* *4. *P =* *0.022; **P =* *0.015; ***P =* *0.044. Error bars indicate SEM.F CWR22 xenografts were grown in nude mice, and tumors were collected at different times after castration. The association between ATF4 and STAMP2 in the tumor samples was determined by Western blot analysis.G The MSKCC Prostate Oncogenome cDNA microarray dataset was obtained from the cBio Cancer Genomics Portal. Analysis was performed as described in Materials and Methods. The expression levels of *STAMP2* and *ATF4,* as well as two ATF4 target genes (*ASNS* and *SLC7A11*), are presented.
Source data are available online for this figure.

To probe the possible signaling pathways which STAMP2 may affect, we performed global gene expression profiling in LNCaP cells upon STAMP2 knockdown compared with control cells. Global gene expression data revealed that activating transcription factor 4 (*ATF4*) expression and that of its target genes (e.g. *ASNS* and *SLC7A11*) were significantly reduced upon STAMP2 knockdown (Supplementary Fig S3A). This was validated by quantitative PCR and Western blot analysis (Fig6A and B). Similar effects of STAMP2 loss on ATF4 expression were observed in LNCaP cells and 22Rv1 cells stably expressing STAMP2 shRNA (Fig6C and D), while ectopic expression of STAMP2 in LNCaP cells induced ATF4 expression (Supplementary Fig S3B). Consistently, in the xenograft tumors formed by 22Rv1 cells with STAMP2 knockdown (Fig5C), expressions of *ATF4* and its target gene *ASNS* were significantly reduced (Fig6E). In addition, ATF4 expression was significantly increased in relapsed CWR22 xenograft tumors, in parallel with a significant increase in STAMP2 expression (Fig6F).

To assess the relevance of these findings to human PCa, we next examined possible correlation of *STAMP2* and *ATF4* expression in a PCa gene expression profile dataset (Taylor *et al*, 2010). Hierarchical clustering analysis was performed, and subgroups showing higher or lower *STAMP2* expression were used for further analysis. As shown in Fig6G, there was a significant positive correlation between *STAMP2* and *ATF4* expression (*R* = 0.4015, *P* < 0.00001), as well as established ATF4 target genes *ASNS* and *SLC7A11*. Similar results were obtained in two independent cohorts (Supplementary Fig S3C). Taken together, these data establish that STAMP2 is involved in regulating ATF4 expression.

### STAMP2 increases PCa cell growth by regulating ATF4 expression

**Figure 7:**
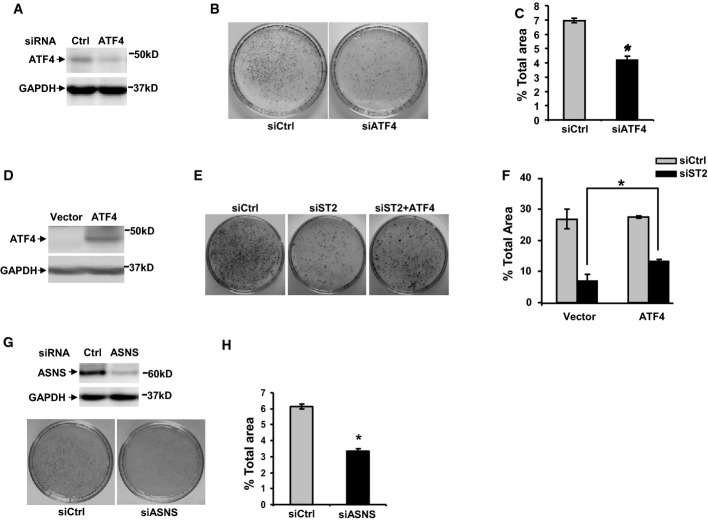
STAMP2 affects PCa cell growth through regulating ATF4 expression
A LNCaP cells were transfected with either control or ATF4 siRNA. Two days after transfection, cells were harvested and whole-cell lysates were made and used in Western blot analysis.B LNCaP cells were transfected with either control or ATF4 siRNA and were cultured for 2 weeks. The colonies formed were stained and photographed.C The area covered by the colonies on each plate in (B) was quantified and represented as percentage of the total area of the plate. *P =* *0.0023.D Cell lysates of LNCaP cells stably expressing an empty vector (Vector) or a vector expressing ATF4 (ATF4) were prepared and subjected to Western blot analysis with the indicated antisera.E After transfection with the indicated siRNAs, cells from (D) were cultured for 2 weeks. The colonies formed were stained and photographed.F The area of the colonies in (E) was quantitated using an image analysis system and represented as percentage of the total area of the plate. *P =* *0.024.G LNCaP cells were transfected with either control (Ctrl) or ASNS siRNA. The cells were then cultured for 2 weeks. The colonies formed were stained and photographed. Knockdown of ASNS was confirmed by Western blot analysis shown at the top.H Quantification of the data shown in (G). *P =* *0.0087.
Data information: In (C, F, and H), Student's *t*-test was used to analyze the statistical significance, *n *=* *3. Error bars indicate SD. Source data are available online for this figure.

ATF4 is an ER stress-, metabolic stress-, and oxidative stress-inducible transcription factor which has key roles in antistress responses (Ye & Koumenis, 2009; Lewerenz & Maher, 2011). Given the above data, we investigated whether ATF4 is involved in STAMP2-mediated proliferative effects on PCa cells. Upon ATF4 knockdown in LNCaP cells (Fig7A), cell growth was significantly decreased (Fig7B and C), indicating that similar to STAMP2, ATF4 is involved in PCa cell growth. We then investigated whether re-expression of ATF4 could reverse cell growth inhibition mediated by STAMP2 knockdown. To that end, LNCaP cells stably expressing an empty vector or a vector expressing ATF4 were established by lentivirus delivery (Fig7D). These cells were then transfected with either control or STAMP2 siRNA and then allowed to grow to form colonies. As shown in Fig7E and F, STAMP2 siRNA-mediated decrease in colony formation was significantly reversed upon ectopic ATF4 expression. This suggests that STAMP2 effects in PCa cells are mediated, at least in part, through ATF4. A target gene of ATF4, *ASNS*, was recently shown to mediate the prosurvival effect of ATF4 in solid tumor cells (Ye & Koumenis, 2009). To evaluate whether this is the case in PCa, we knocked down ASNS in LNCaP cells by siRNA which resulted in a significant reduction in cell growth (Fig7G and H). These data further confirmed the prosurvival role of ATF4 signaling in PCa cells.

### STAMP2 has ferrireductase activity and increases ROS levels

**Figure 8:**
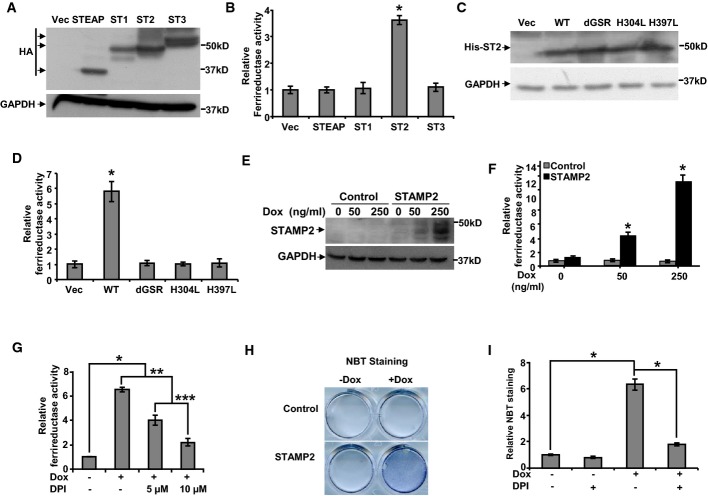
ROS generation by STAMP2 depends on its ferrireductase activity
A 293T cells were transfected with plasmids expressing either an empty vector (Vec) or vectors expressing HA-tagged STEAP, STAMP1 (ST1), STAMP2 (ST2), or STAMP3 (ST3). Whole-cell extracts were prepared and subjected to Western blot analysis using anti-HA antibody or GAPDH as a loading control.B Relative ferrireductase activities were measured in the cells expressing the different constructs used in (A) as described in Materials and Methods. *P < 0.0001.C 293T cells were transfected with different plasmids expressing either an empty vector (Vec) or vectors expressing His-tagged wild-type STAMP2 (WT) or three STAMP2 mutants (dGSR, H304L, or H397L) as indicated. Whole-cell extracts were prepared and used in Western blot analysis with anti-His antibody or GAPDH antibody as a loading control.D Relative ferrireductase activities were measured in the cells from (C) as described in Materials and Methods. *P =* *0.0003.E 293T cell line with Dox-inducible STAMP2 expression or vector control was generated. Western blot analysis confirmed STAMP2 expression in a Dox-inducible manner.F The cells from (E) were either left untreated or treated with increasing amounts of Dox, and ferrireductase activity was determined. *P < 0.0001.G The cells with Dox-inducible STAMP2 expression from (E) were either left untreated or treated with Dox for 48 h. Prior to ferrireductase activity measurement, the cells were treated with or without DPI as indicated for 1 h. *P < 0.0001; **P =* *0.0012; ***P =* *0.048.H The cells from (E) were treated with 100 ng/ml Dox for 2 days. Oxidative stress was then measured by NBT staining, and the stained cells were photographed. There were equal numbers of cells on the plates for −/+ Dox as shown in Supplementary Fig S4.I The cells with Dox-inducible STAMP2 expression from (E) were either left untreated or treated with Dox for 48 h. Then, the cells were treated with or without 10 μM DPI before being subjected to NBT staining. *P < 0.0001.
Data information: In (B, D, F, G, and I), Student's *t*-test was used to analyze the statistical significance, *n *=* *3. Error bars indicate SD. Source data are available online for this figure.

Previous studies have shown that ATF4 expression is induced by oxidative stress (Cullinan & Diehl, 2006). Since STAMP2 is linked to ATF4 expression, oxidative stress may be affected by intracellular iron levels (Knobel *et al*, 2006). Furthermore, mouse Stamp2 has iron reductase activity in 293T cells (Ohgami *et al*, 2006). We thus hypothesized that STAMP2 expression may contribute to intracellular ROS generation and thus ATF4 expression. We first assessed the iron reductase activity of human STAMP proteins ectopically expressed in 293T cells (Fig8A). In contrast to the mouse STAMPs, only STAMP2 displayed iron reductase activity in the human family (Fig8B). Mutagenesis of the putative FAD (dGSR) or heme-binding sites (H304L or H397L) abolished STAMP2 iron reductase activity (Fig8C and D) consistent with findings on mouse Stamp2 (Ohgami *et al*, 2005, 2006). To follow up on these findings, we generated a 293T cell line with doxycycline (Dox)-inducible STAMP2 expression (Fig8E) in which iron reductase activity was induced in a Dox-dependent manner (Fig8F). Moreover, the oxidoreductase inhibitor diphenyleneiodonium sulfate (DPI), which inhibits flavoproteins, suppressed STAMP2 activity in a dose-dependent manner (Fig8G). These data show that human STAMP2 has ferrireductase activity.

**Figure 9:**
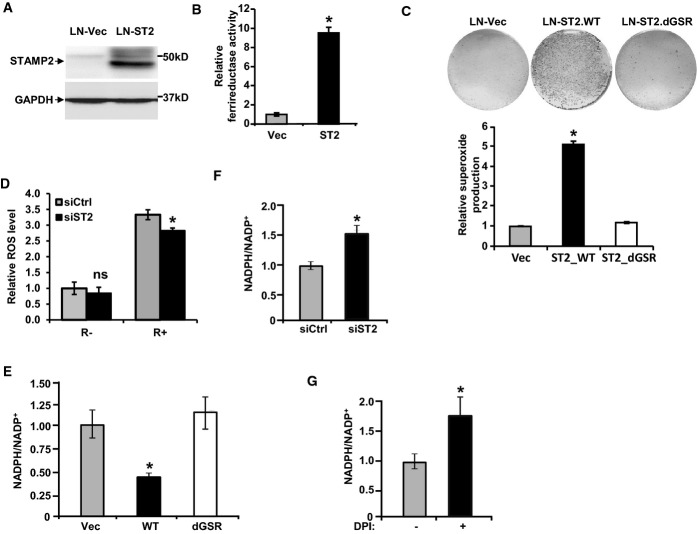
STAMP2 increases intracellular ROS by decreasing intracellular NADPH levels
A LNCaP cells stably expressing an empty vector (LN-Vec) or a vector expressing STAMP2 (LN-ST2) were established by lentivirus delivery. Cell lysates were prepared and subjected to Western blot analysis with the indicated antisera.B Relative ferrireductase activities were measured in the cells described in (A). *P =* *0.0003.C NBT staining was performed in LNCaP cells stably expressing either an empty vector (LN-Vec) or a vector expressing wild-type STAMP2 (LN-ST2/WT) or a STAMP2 mutant (LN-ST2/dGSR) as indicated. The stained cells were then photographed and quantified. *P =* *0.004.D LNCaP cells were transfected with either scrambled siRNA (siCtrl) or siRNA against STAMP2 (siST2) and were cultured in the presence of 10 nM R1881 or vehicle for 2 days. Intracellular ROS levels were then measured by CellROX reagent staining. *P =* *0.002. ns, not significant.E LNCaP cells stably expressing an empty vector (Vec), a vector expressing wild-type STAMP2 (WT), or a STAMP2 mutant (dGSR) were cultured, and the NADPH/NADP^+^ ratio was determined as described in Materials and Methods. *P =* *0.015.F LNCaP cells were transfected with either control siRNA (siCtrl) or STAMP2 siRNA (siST2). Cells were then cultured for 2 days and harvested, and the NADPH/NADP^+^ ratio was determined as above. *P =* *0.026.G LNCaP cells stably expressing wild-type STAMP2 were treated with or without DPI (1 μM) for 4 h, harvested, and used in the NADPH/NADP^+^ assay. *P =* *0.018.
Data information: Student's *t*-test was used to analyze the statistical significance, *n *=* *3. Error bars indicate SD.

We next assessed whether the iron reductase activity of STAMP2 is associated with oxidative stress. Ectopic STAMP2 expression in 293T cells significantly enhanced ROS levels (Fig8H, Supplementary Fig S4). Furthermore, the elevation in ROS upon STAMP2 expression was abolished by DPI (Fig8I). Although DPI is a widely used inhibitor of ROS generating oxidoreductases, under some conditions, it has been shown to have inhibitory effects on other flavoproteins that can affect ROS levels in the cell (Riganti *et al*, 2004). Thus, to further confirm the dependence of STAMP2 ferrireductase activity for ROS production, we used the dGSR mutant of STAMP2 and compared its ability to generate ROS with that of wild-type STAMP2. As shown in Fig9C, inactivation of STAMP2 ferrireductase function completely abolished superoxide production. To further evaluate the involvement of STAMP2 in ROS generation, we used the antioxidant N-acetyl cysteine (NAC) which effectively inhibited STAMP2-induced NBT reduction (Supplementary Fig S5). These data show that STAMP2-mediated increase in oxidative stress requires its ferrireductase activity.

To determine whether oxidative stress also induces ATF4 expression in PCa cells, we used menadione, an agent that increases intracellular ROS levels (Cafe *et al*, 1995). As shown in Supplementary Fig S6A and B, ATF4 mRNA and protein levels were significantly increased in LNCaP cells in response to menadione, indicating that oxidative stress regulates ATF4 expression in PCa cells. We then determined whether STAMP2-induced ATF4 expression in PCa cells could be inhibited by an antioxidant. To that end, LNCaP cells ectopically expressing STAMP2 or vector control were either left untreated or treated with N-acetyl cysteine (NAC) and ATF4 protein levels were determined by Western blot analysis. As shown in Supplementary Fig S6C, NAC treatment completely inhibited STAMP2-induced ATF4 expression. Consistently, inducible expression of STAMP2 in 22RV1 cells increased ATF4 expression which was blocked in response to NAC treatment (Supplementary Fig S6D). In addition, the ferrireductase inactive STAMP2 mutant, which cannot increase intracellular ROS levels, did not affect ATF4 expression (Supplementary Fig S6E). These data show that oxidative stress induced by STAMP2 is involved in increasing ATF4 expression in PCa cells.

### STAMP2 depletes intracellular NADPH

We have recently shown that mouse Stamp2 is involved in NADPH homeostasis in macrophages and regulates inflammatory responses (ten Freyhaus *et al*, 2012). In addition, NADPH levels are inversely correlated with ROS levels (Ying, 2008). We thus determined whether STAMP2 regulates NADPH levels in PCa cells which may be linked to its ability to increase ROS. Ectopic expression of STAMP2 in LNCaP cells (Fig9A) led to robust increases in iron reductase activity and superoxide production (Fig9B and C). In addition, STAMP2 knockdown decreased ROS production in PCa cells (Fig9D, Supplementary Fig S7). Furthermore, expression of wild-type STAMP2, but not an iron reductase-deficient STAMP2 mutant, resulted in a significant decrease in the NADPH/NADP^+^ ratio (Fig9E). Consistently, STAMP2 knockdown in LNCaP cells (Fig9F), as well as the flavoenzyme inhibitor DPI (Fig9G), also significantly increased the NADPH/NADP^+^ ratio. These data suggest that STAMP2 iron reductase activity is required for modulation of the intracellular redox state and ROS levels.

### Therapeutic targeting of STAMP2 by nanoliposomal siRNA reverses PCa growth *in vivo*

**Figure 10:**
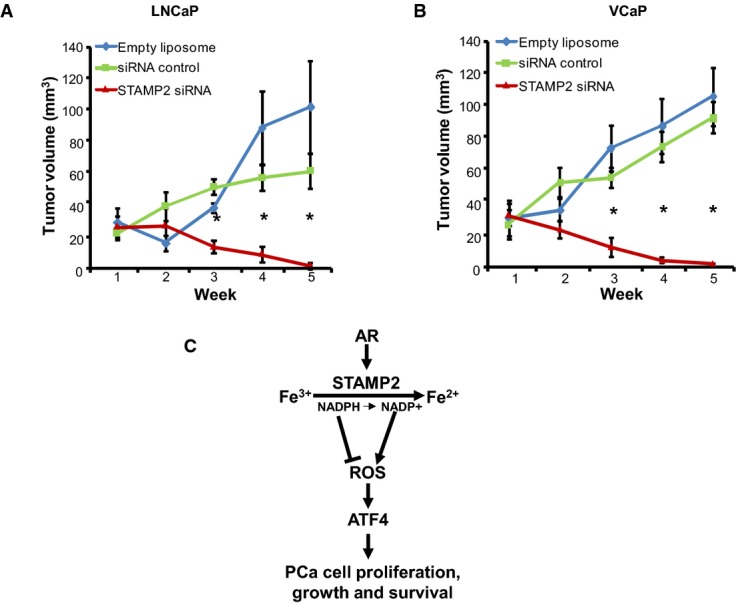
Targeting STAMP2 in preclinical models of PCa results in tumor regression
A LNCaP cells were implanted subcutaneously into nu/nu mice. Once tumors reached 5 mm in size, mice (*n* =* *5 per group) were given nanoliposome-encapsulated control siRNA or STAMP2 siRNA as described in Materials and Methods. Tumor volumes were measured at the indicated time points. *P =* *0.001.B Same experiment as in (A), but VCaP cells were used instead. *P =* *0.001.C A model for STAMP2 effects on growth and survival of PCa cells. Once activated, AR increases STAMP2 expression. STAMP2 catalyzes reduction of Fe^3+^ to Fe^2+^, also depleting NADPH levels, which increases ROS. Increased ROS then activate ATF4 expression which in turn activates target genes that adapt PCa cells to oxidative stress resulting in cell proliferation and survival.
Data information: Student's *t*-test was used to analyze the statistical significance between mice (*n* =* *5 per group) treated with control siRNA vs STAMP2 siRNA. Error bars indicate SEM.

The data presented above showed that STAMP2 promotes proliferation, colony formation, anchorage-independent growth, survival, and tumor growth of PCa cells. However, the role of STAMP2 in tumorigenesis and the therapeutic potential of its knockdown, if any, are not known. To assess this possibility, *STAMP2* was silenced using systemically administered nanoliposomal siRNA in nude mice carrying xenografted tumors of LNCaP or VCaP cells. This strategy has successfully been used in similar experiments in various cancer models, including PCa (e.g. Landen *et al*, 2005; Nick *et al*, 2011; Jin *et al*, 2013). As shown in Fig10A and B, whereas tumors continued to grow rapidly in mice injected with the empty or control siRNA-loaded nanoliposomes, there was a dramatic and time-dependent reversal of tumor size upon injection of nanoliposomes containing STAMP2 siRNA in both tumor models reaching regression of > 95% within 5 weeks. The *in vivo* knockdown efficacy was confirmed by qPCR analysis in tumor tissues collected at the end of the experiments (Supplementary Fig S8). These data establish that targeting STAMP2 can profoundly reverse tumor growth in preclinical models of human PCa.

## Discussion

Here, we have demonstrated that STAMP2 promotes PCa tumorigenesis and progression by serving as a proliferation and survival factor and thus may serve as a potential therapeutic target. Our data also reveal a novel mechanism through which androgen signaling increases ROS levels in PCa cells through induction of STAMP2 expression and its oxidoreductase activity.

One of the factors that has been implicated in PCa is oxidative stress (for a review, see Paschos *et al*, 2013). Several lines of evidence have documented an altered prooxidant–antioxidant status in PCa. For example, early studies found that androgen signaling induced a shift toward a prooxidant state in PCa cells (Ripple *et al*, 1997; Sun *et al*, 2001). Consistently, it was shown that androgen ablation reduced oxidative stress through down-regulation of NADPH oxidase expression (Tam *et al*, 2003). Furthermore, increased ROS and DNA damage have been documented in PCa (Frohlich *et al*, 2008). In keeping with these findings, somatic mutations that inactivate the glutathione S-transferase P1 (*GSTP1*) gene have been identified in the majority of PCa cases (Nelson *et al*, 2004). Despite these data, our knowledge has been limited on the molecular mechanisms that regulate ROS levels in PCa cells. The results presented herein, demonstrating the direct involvement of STAMP2 in ROS production in PCa cells, provide new insight into these events.

The ability of STAMP2 to increase ROS levels is likely via increasing ferrous iron levels in the cells which is then used as a redox intermediate (the electron donor) for free radical production (Okada, 1996). In addition, NADPH, which normally inhibits ROS (Ying, 2008), is used up during the ferrous iron production by STAMP2, thus further increasing ROS levels. It is also possible that STAMP2 helps increase ROS levels in the cell through suppression of the antioxidant defense system.

It was previously shown that the activation of the monoamine oxidase system through the enzyme spermidine/spermine N1-acetyltransferase (SSAT) may increase ROS levels in PCa cells (Basu *et al*, 2009; Mehraein-Ghomi *et al*, 2010). Inhibition of monoamine oxidases, even at levels that are tenfold higher than that required to inhibit SSAT, did not affect STAMP2-induced ROS production in LNCaP cells (data not shown) indicating that STAMP2- and SSAT-mediated oxidative stress pathways in PCa cells are independent.

To elucidate the molecular mechanisms responsible for the proliferative effect of STAMP2, we used gene expression profiling. We found that *ATF4* is one of the genes whose expression was most significantly reduced upon STAMP2 knockdown. ATF4 is induced by various cellular stresses, including metabolic, oxidative, and ER stress, and is an important regulator of gene expression that is involved in amino acid metabolism and transport, antistress response (such as restoration of normal ER function and redox balance), and cell survival (Harding *et al*, 2003; Ye & Koumenis, 2009). Elevated expression of ATF4 in cancer cells has been associated with resistance to some chemotherapeutic drugs, such as DNA-damaging agents and proteasome inhibitors (Rzymski *et al*, 2009). A recent study revealed that the GCN2-PERK-eIF2α-ATF4 pathway is required for survival and proliferation of cancer cells in response to nutrient deprivation (Ye *et al*, 2010). A recent study suggested that L-type amino acid transporters, which are target genes of ATF4, promote PCa cell survival through maintaining amino acid supplementation (Wang *et al*, 2013); however, the exact role of ATF4 in PCa cells has not been determined to date. Here, we found that similar to depletion of STAMP2, ATF4 knockdown inhibited PCa cell growth (Fig7B and C). Furthermore, ectopic expression of ATF4 partially reversed growth inhibition that is induced by STAMP2 knockdown (Fig7E and F). These data suggest that ATF4 directly contributes to the proliferative activities of STAMP2 in PCa cells.

Consistent with the *in vitro* and *in vivo* data, STAMP2 expression was significantly increased in human PCa compared with normal prostate (Fig1). In addition, STAMP2 levels correlated with tumor grade and neoadjuvant hormone therapy response (Fig4). A limitation of these data is that almost all patients in these cohorts were Caucasian, and thus, additional studies will be required to assess whether our findings can be extended to all men with PCa. Furthermore, analysis of independent cohorts and even larger number of patients is desirable.

Based on the data presented herein, we suggest the following model as to how STAMP2 influences PCa growth (Fig10C): Androgens activate the AR that increases STAMP2 expression. Through its iron reductase activity, STAMP2 reduces Fe^3+^ to Fe^2+^ and at the same time consumes available NADPH. Both increased Fe^2+^ and decreased NADPH then increase intracellular ROS levels. ROS in turn activate the pathway that increases ATF4 expression which then activates expression of downstream targets that are involved in PCa growth and progression. Other pathways may also be involved in the prosurvival function of STAMP2 in PCa cells, and further exploration is required.

In addition to STAMP2 effects on PCa growth and survival, and its correlation with tumor grade and response to hormone therapy (Figs1 and 4), STAMP2 targeting by systemic administration of nanoliposome-encapsulated siRNA resulted in profound tumor regression in two independent preclinical models of human PCa (Fig10A and B). These findings suggest that STAMP2 could have utility in the clinic as a biomarker, as well as a therapeutic target, including in advanced disease.

## Materials and Methods

### Cell culture

293T cells were obtained from the American Type Culture Collection (LGC Standards) and routinely maintained in standard growth conditions. LNCaP cells and VCaP cells (ATCC, LGC standard) were routinely maintained in RPMI 1640 culture medium supplemented with 10% fetal bovine serum (FBS), 5 mg/ml penicillin/streptomycin, and 2 mM l-glutamine (Invitrogen) at 37°C in a humidified atmosphere with 5% CO_2_. All the cell cultures were performed under these conditions unless specifically indicated. Their response, or lack thereof, to androgen treatment for growth, as well as in reporter assays, was determined as a way to authenticate the PCa lines. All cell lines were free of mycoplasma contamination.

### Immunohistochemistry

This study was approved by the Regional Ethics Committee, REK Sør-Øst (S-07443a), and material from still living patients was included after their written consent. The prostate tissue microarrays (TMAs) for Fig1 were previously described (Klokk *et al*, 2007; Wang *et al*, 2010). After deparaffinization, antigen retrieval was done by autoclaving at 121°C for 10 min in 10 mM citrate buffer (pH 6.4). The affinity purified STAMP2 antibody (Proteintech Group, Inc.) was used at a dilution of 1:50 for 1 h at room temperature. The Supersensitive Detection kit (Biogenex) was used for antigen detection (Klokk *et al*, 2007).

For the neoadjuvant hormone therapy (NHT) and PSA recurrent samples, total of 194 prostate cancer specimens were obtained from Vancouver Prostate Centre Tissue Bank. The H&E slides were reviewed, and the desired areas were used to construct TMAs (Beecher Instruments, MD, USA). All specimens were from radical prostatectomies except 12 CRPC samples that were obtained through transurethral resection of prostate (TURP). Details of the material are presented in Supplementary Table S1. IHC was conducted by Ventana autostainer model Discover XT (Ventana Medical System, Tuscan, Arizona) with enzyme labeled biotin streptavidin system and solvent-resistant DAB Map kit.

### Scoring and statistical analysis of TMAs

For scoring, values on a four-point scale were assigned to each immunostain. Descriptively, one represents no apparent staining or very weak level of staining, two represents a faint or focal, questionably present stain, three represents a stain of convincing intensity in a minority of cells, and four represents a stain of convincing intensity in a majority of cells. SPSS 10.0 software was used for IHC statistical analysis. To compare STAMP2 expression between benign tissue and malignant tissue, Mann–Whitney *U*-test was applied. The Kruskal–Wallis test was used for the analysis of correlation between STAMP2 expression and tumor grade.

### Correlation analysis of *ATF4* and *STAMP2* expression in PCa samples

A PCa gene expression profile dataset available at the cBioCancer Genomics Portal (GSE21032) (Taylor *et al*, 2010) was used for this analysis. The samples (*n* = 150) were aligned by the expression level of *STAMP2*. The subgroups showing the highest (*n* = 30) and the lowest (*n* = 30) *STAMP2* expression were then used for possible correlation with the expression of *ATF4* and two ATF4 target genes, *ASNS* and *SLC7A11*. The results are displayed in a heat map generated by the GSEA program. The statistical significance was determined by *t*-test. The correlation test was further applied to PCa samples of other two datasets, GSE35988 (*n* = 59) (Grasso *et al*, 2012) and GES6919 (*n* = 62) (Yu *et al*, 2004), using the same methodology.

### Plasmids

Human STEAP, STAMP1, STAMP2, and STAMP3 cDNAs were cloned into SR-alpha vector (kind gift from Tiliang Deng) to introduce an N-terminal HA tag. STAMP2 cDNA was also cloned into pcDNA4/HisMax-TOPO vector (Invitrogen). STAMP2 mutants were generated from pcDNA4-HisMax-STAMP2 with GeneTailor™ Site-Directed Mutagenesis System (Invitrogen). Inducible lentiviral vector for overexpressing STAMP2 was generated from pGIPZ or pTRIPZ vectors (Open Biosystems, Thermo Fisher Scientific, Inc.) by removing shRNA coding fragment and replacing the GFP ORF (pGIPZ) or RFP ORF (pTRIPZ) with the STAMP2 ORF. Precision lentiORF ATF4 and Precision lentiORF RFP vectors were obtained from Open Biosystems, Thermo Fisher Scientific, Inc. Inducible adenoviral vector for overexpressing STAMP2 was generated as described previously (Klokk *et al*, 2007).

### Ectopic expression of STAMP2

To obtain cells stably expressing STAMP2, lentivirus-based vectors were generated as described above and virus was produced with Trans-Lentiviral™ Packaging kit (Thermo Fisher Scientific). LNCaP cells or 293T cells were transduced with lentivirus and selected with puromycin.

### NADPH measurements

Measurement of NADPH and NADP^+^ was performed using a previously described enzymatic cycling method (Ros *et al*, 2012). Briefly, cells from a 6-cm dish were lysed in 400 μl of extraction buffer (20 mM nicotinamide, 20 mM NaHCO_3_, 100 mM Na_2_CO_3_) and centrifuged. Twenty microliter of the supernatant of each sample was loaded to a 96-well plate for determining total NADP level. To determine NADPH level, 150 μl of the supernatant was incubated at 60°C for 30 min to decompose NADP^+^, and 20 μl of the sample was loaded to 96-well plate. Then, 160 μl of NADP-cycling buffer (100 mM Tris–HCl pH 8.0, 0.5 mM thiazolyl blue, 2 mM phenazine ethosulfate, 5 mM EDTA) containing 1.3 U of G6PD was added to the 96-well plate containing 20 μl of the samples. After a 1-min incubation in the dark at 30°C, 20 μl of 10 mM glucose 6-phosphate (G6P) was added to the mixture, and the change in absorbance at 570 nm was measured every 30 s for 4 min at 30°C with a microplate reader. The concentration of NADP^+^ was calculated by subtracting [NADPH] from [total NADP].

### Cell proliferation assay

Cells were cultured for the indicated times, trypsinized, stained with trypan blue, and counted using a hemocytometer. The experiments were repeated at least three times with consistent results. Alternatively, cell proliferation was determined by the use of Cell Counting kit-8 from Sigma-Aldrich.

### Colony formation assay

Cells were trypsinized, seeded on dishes, and cultured for 14 days. The cells were then fixed and stained with 0.1% crystal violet, and the total area covered by the colonies was measured.

### Soft agar assay

Low-melting (LE) agarose was purchased from LONZA. Two milliliter of 0.6% LE agarose (in culture medium) was layered in the bottom of 6-cm dishes. A total of 5,000 cells suspended in 2 ml of 0.35% LE agarose (in culture medium) at 37°C were then plated on top of the first layer. The cells in the top layer were fed with 100 μl culture medium every 3 days. After 14 days, the cells were stained with 0.005% crystal violet (in PBS) and then photographed. The number of colonies (> 30 pixels) on each plate was counted using an imaging system (Syngene).

### Cell cycle analysis

Cells were synchronized at the G0/G1 phase by serum starvation for 48 h and then released into cell cycle by re-addition of 10% FBS. After 24 h, cells were trypsinized, washed with PBS, and then fixed in 70% ice-cold ethanol for 2 h at 4°C. To assess the cell cycle profile, fixed cells were treated with RNase (Sigma-Aldrich), stained with propidium iodide (PI) (Sigma-Aldrich), and analyzed by flow cytometry using the CellQuest software.

### Apoptosis assays

To detect pro-apoptotic factor-induced apoptosis, cells were treated with either 50 ng/ml tumor necrosis factor (TNF)-related apoptosis-inducing ligand (TRAIL; Enzo Life Sciences) or 20 μM LY294002 (Invitrogen) for 24 h or both agents for 6 h. Extent of apoptosis was then detected by terminal deoxynucleotidyl transferase-mediated dUTP nick-end labeling (TUNEL) and flow cytometry as described by the manufacturer's instructions (Roche).

### Quantitative PCR

RNA extraction, cDNA synthesis, and quantitative PCR were performed as described previously (Klokk *et al*, 2007). PCR primer sequences are available upon request. A standard curve made from serial dilutions of cDNA was used to calculate the relative amount of the different cDNAs in each sample. The values were normalized to the relative amount of the internal standard GAPDH or TBP. The experiments were performed in triplicate with consistent results.

### Western blot analysis

Fractionation of subcellular proteins was done by using Subcellular Protein Fractionation Kit for cells (Thermo Fisher Scientific Inc.). Western blot analysis was performed by standard methods. Results shown are representative of at least two independent experiments. Antibodies specific for cleaved caspase-3 (#9664), cleaved PARP (#5625), and p21CIP1 (#2947) were from Cell Signaling; antibody for STAMP2 (11944-1-AP) and ATF4 (10835-1-AP) was from Proteintech Group Inc.; and antibodies for PCNA (P8825) and GAPDH (G8795) were from Sigma. Antibody for STAMP1 was described previously (Wang *et al*, 2010). All antibodies were used at a dilution of 1:1,000, except for GAPDH (1:5,000).

### Small interfering RNA-mediated knockdown

LNCaP cells were transfected with ATF4 siRNA (Thermo Scientific Dharmacon) or the other siRNAs (Qiagen) using Oligofectamine (Invitrogen) according to the manufacturer's recommendations. The sequences of STAMP2 siRNAs are 5′-AAUGCAGAGUACCUUGCUCAU-3′ and 5′-UAUCCAUCACUCUUUGCUUGG-3′. The SMARTpool siRNA from (DharmaconGE) was used to knock down ATF4 or ASNS.

### Short hairpin RNA-mediated knockdown

Lentiviral pLKO.1 short hairpin RNA (shRNA) vectors targeting human STAMP2 and non-silencing control vector were purchased from Open Biosystems (Thermo Fisher Scientific, Inc.). Lentivirus particles were produced in 293T cells according to the manufacturer's instructions. LNCaP cells were transduced with the lentiviral particles following puromycin selection (1 μg/ml) for 10 days. The cells stably expressing shRNA were pooled and maintained in medium containing puromycin (0.2 μg/ml).

### Ferrireductase activity assay

Ferrireductase assays were conducted as previously described (Ohgami *et al*, 2005, 2006). Briefly, cells were cultured in 12-well plates to 80–90% confluence. After three washes with phosphate-buffered saline (PBS, pH 7.2), the cells were incubated in dark in iron uptake buffer (IUB: 25 mM MES, 25 mM MOPS, 140 mM NaCl, 5 mM glucose, 5.4 mM KCl, 1.8 mM CaCl_2_, 800 μM MgCl_2_) containing 50 μM ferric nitrilotriacetate (Fe^3+^-NTA) and 200 μM ferrozine for 30–60 min. The production of ferrous iron was determined by monitoring the increase in absorbance at *λ* = 562 nm using a spectrophotometer. The experiments were repeated at least three times with consistent results.

### Intracellular ROS assays

Nitro blue tetrazolium (NBT) staining: NBT staining was carried out as described previously (Serrander *et al*, 2007). The amount of reduced NBT was quantified by spectrophotometry. The experiments were repeated at least three times with consistent results.

CellROX staining: Cells were stained with 500 nM CellROX Green reagent (Life Technologies) for 30 min and then trypsinized. Intracellular fluorescence intensity was measured using the FL1 channel in a BD FACSCalibur flow cytometer. Data were analyzed using CellQuest software. The experiments were repeated at least three times with consistent results.

### Xenograft experiments in SCID or nude mice

For injection into mice, 3 million LN-shST2 or LN-Scr cells were suspended in 50 μl RPMI 1640 and mixed with 50 μl Matrigel (BD Biosciences). The mixtures were then subcutaneously implanted in random into NOD/SCID IL2R gamma null mice in both hind flanks. Tumor size was measured weekly in two dimensions with calipers, and the tumor volume V was calculated according to the formula: V = W2 × L × 0.5, where W and L are tumor width and length, respectively. No blinding was carried out. Same procedure was conducted in nude mice (BALBC Nu/Nu, 5 weeks of age) for 22Rv1 cells stably expressing shRNA control or shRNA targeting STAMP2. All procedures on animals, including mouse strain, animal sex, age, number of animals allowed to use, and housing details, were approved by the National Animal Research Authority and were conducted according to the regulations of the Federation of European Laboratory Animals Science Association.

### Nanoliposomal siRNA targeting in PCa xenografts

*In vivo* therapeutic targeting of STAMP2 by systemically administered nanoliposomal siRNA was carried out as described previously (Landen *et al*, 2005; Nick *et al*, 2011; Shao *et al*, 2012; Stone *et al*, 2012). Athymic male nu/nu mice (5 weeks old) were used in random to inject LNCaP or VCaP cells (2 × 10^6^) subcutaneously into the right flank of each mouse (six mice per group) with 10% matrigel. Once tumors reached 3–5 mm in size, mice were given nanoliposome-encapsulated non-silencing control siRNA or STAMP2 siRNA 150 μg/kg (∽4 μg siRNA/mouse), twice a week from the tail vein in 100 μl saline. At the indicated time points, the tumor volumes were measured weekly in two dimensions with calipers and the tumor volume V was calculated according to the formula: V = W^2^ × L × 0.5, where W and L are tumor width and length, respectively. No blinding was carried out. After 5 weeks of nanoliposomal siRNA treatment, tumors were harvested.

### Statistical analysis

Mean and standard deviation values were calculated using Microsoft Excel software. The treatment effects in each experiment were compared by two-sided *t*-test. One-way analysis of variance (ANOVA) with a *post hoc* test was used for comparing multiple independent variables. Differences between groups were considered significant at *P* < 0.05. All experiments were repeated three times. In animal experiments, we used 5–6 animals per group. For all *in vitro* experiments, the results of one representative experiment are shown as mean values ± SD or ± SEM as indicated in the respective figure legends.

## Supporting Information

Source Data for Supplementary Figure S6

Review Process File
