# Supplementary material for: STAMP2 increases oxidative stress and is critical for prostate cancer
Source: EMBO Mol Med. 2015 Feb 13;7(3):315–31. doi: 10.15252/emmm.201404181 (PMC4364948; doi:10.15252/emmm.201404181)
Supplement: Supplementary file 1 [file emmm0007-0315-sd1.docx]

**Supplementary Materials:**

1. **Table S1**
2. **Supplemenatry Figure S1-S8**

**Supplementary Table S1:**

**Table S1.** Detailed information on the patients and the material used in constructing the TMAs.

| **Gleason Graded TMA-2009** | **Pathology** | **#Patients** | **#Cores** | **Treatment** |
| --- | --- | --- | --- | --- |
|  | BPH/PIN | 23 | 46 | None |
|  | Gleason 6-7 | 35 | 70 | None |
|  | Gleason 7-8 | 24 | 48 | None |
|  | Gleason 9-10 | 6 | 12 | None |
|  | **Total** | **88** | **176** |  |
|  |  |  |  |  |
| **NHT TMA-2009** | G3 | 7 | 14 | None |
|  | G4 | 3 | 6 | None |
|  | G5 | 4 | 8 | None |
|  | TR Pattern | 5 | 10 | 1M |
|  | TR Pattern | 3 | 6 | 1.5M |
|  | TR Pattern | 3 | 6 | 2M |
|  | TR Pattern | 5 | 10 | 3M |
|  | TR Pattern | 8 | 16 | 5-6M |
|  | TR Pattern | 18 | 36 | 7-8M |
|  | TR Pattern | 16 | 32 | 9-12M |
|  | TR Pattern | 16 | 32 | CRPC |
|  | **Total** | **88** | **176** |  |
|  |  |  |  |  |
| **CRPC/TURP-2009** | CRPC | 12 | 24 | N/A |
|  | Benign |  | 2 | N/A |
|  | Cancer | 3 | 2 | N/A |
|  | LN Mets |  | 2 | N/A |
|  | Native | 3 | 6 | N/A |
|  | **Total** | **18** | **36** |  |
|  |  |  |  |  |
| **Total # of Patients: 194** |  |  |  |  |
| **Total # of Cores: 388** |  |  |  |  |

**Supplementary Figure S1:**

**Figure S1. siRNA mediated STAMP2 knockdown in LNCaP and VCaP cells**.

A LNCaP cells were cultured in RPMI 1640 medium containing 10% CT-FBS and treated with (R+) or without (R-) 1 nM R1881 for 24 h before transfection with indicated siRNAs. The cells were then cultured 48 h before RNA extraction and qPCR analysis. Student’s t-test was used to analyze the significance, N=3. *, P < 0.0001. Error bars indicate SD.

B VCaP cells were cultured in RPMI 1640 medium containing 10% CT-FBS and treated with or without 1 nM R1881 for 24 h before being transfected with indicated siRNAs. The cells were then cultured 48 h before RNA extraction and qPCR analysis. Student’s t-test was used to analyze the significance, N=3. *, P < 0.0001. Error bars indicate SD.

C-D Quantification of cleaved caspase3 (cCaspase3) and cleaved PARP (cPARP) upon STAMP2 knockdown. LNCaP cells were transfected with either control or two independent STAMP2 specific siRNAs, ST2-1 and ST2-2. 3 days after transfection, cells were treated with either 50 ng/mL TRAIL or 20 μmol/L LY294002 (LY) for 24 h, or both agents for 6 h, and then subjected to Western analysis for cleaved caspase3 and cleaved PARP. Blots from 3 independent experiments were used for quantification using Image J. Student’s t-test was used to analyze the significance, N=3. *, P < 0.05. Error bars indicate SD.. Even though for TRAIL alone treatment, the difference did not reach significance, there was a clear trend for an increase when STAMP2 is knocked down.

**Supplementary Figure S2:**

**Figure S2. STAMP2 knockdown inhibits growth of 22Rv1 cells**.

A STAMP2 expression levels in 22Rv1 cells stably expressing STAMP2 specific or control shRNA were determined by qPCR (upper panel) and Western analyses (lower panel). Student’s t-test was used to analyze the significance, N=3. *, P < 0.0001. Error bars indicate SD.

B Equal number of cells from A were plated and cultured for 10 days. The colonies formed were then stained, photographed and quantified. Representative microscope images are shown on top. Student’s t-test was used to analyze the significance, N=3. *, P < 0.0001. Error bars indicate SD.

**Supplementary Figure S3:**

**Figure S3. STAMP2 and ATF4 expression is correlated *in vitro* and *in vivo***.

A LNCaP cells were cultured in RPMI1640 medium containing 10% CT-FBS in the presence of absence of 1 nM R1881 for 24 h before being transfected with indicated siRNAs. The cells were then cultured 48 h, subjected to RNA extraction and cDNA microarray analysis. The top 50 genes that were downregulated in STAMP2 knockdown cells are shown in the heatmap.

B LNCaP cells were transfected with plasmid DNA expressing 3HA-tagged wild type STAMP2 or empty vector alone. Three days after transfection, the cytosolic fraction (Cyt.) and nuclear fraction (Nucl.) were prepared and subjected to Western analysis with an ATF4 monoclonal antibody (Cell Signaling Technology, #11815) and HA antibody (Sigma-Aldrich, #H9658). HDAC1 and actin were used as loading controls.

C The cDNA microarray data sets of two PCa cohorts (GSE35988 and GSE6919) were analyzed as described in Materials and Methods. The expression levels of *STAMP2* and *ATF4,* as well as two *ATF4* target genes (*ASNS* and *SLC7A11*) are presented. The p value of Pearson correlation between STAMP2 and the rest genes are less than 0.05.

**Supplementary Figure S4:**

**Figure S4.** 293T cells with Dox inducible STAMP2 expression or vector control were treated with or without doxycycline (Dox) for 48 h. The cells were then subjected to NBT staining (see Figure 8H). After photographing for NBT staining, the plate was further stained with 0.01% crystal violet solution to determine the general cell distribution on the plates. Note that some patches of cells came off the plate during the staining procedure.

**Supplementary Figure S5:**

**Figure S5.** STAMP2-mediated ROS production is inhibited by antioxidants. A 293T cell line with Dox inducible STAMP2 expression was treated with 100 ng/ml doxcycline to induce STAMP2 expression. The cells were then treated with or without 50 mM N-acetyl cysteine (NAC) for 2 h before being NBT staining. The data show a representative experiment done in triplicate.

**Supplementary Figure S6:**

**Figure S6. Oxidative stress induced ATF4 expression in LNCaP cells**.

A LNCaP cells were left untreated or treated with 50µM menadione for 4 h. The cells were then subjected to RNA extraction and qPCR analysis. Student’s t-test was used to analyze the significance, N=3. *, P < 0.0001. Error bars indicate SD.

B LNCaP cells were left untreated or treated with 50µM menadione for the indicated times. The cytosolic fraction (C) and nuclear fraction (N) were prepared, and were used in Western analysis of ATF4 expression. HDAC1 was used as a loading control.

C LNCaP cells stably expressing either control or STAMP2 vector were treated with or without 10 mM NAC for 2h. The cytosolic fraction (Cyt.) and nuclear fraction (Nucl.) were prepared and subjected to Western analysis with an ATF4 monoclonal antibody (Cell Signaling Technology, #11815) and STAMP2 antibody (Proteintech Group Inc, #11944-1-AP). HDAC1 and actin were used as loading controls.

D 22Rv1 cells were infected by Ad-STAMP2(HA-tagged) adenovirus and STAMP2 expression was induced by addition of 100 ng/ml doxycyline (Dox) for 24h. The cells were then treated with or without 10 mM NAC for 2h. The cell lysates were prepared and subjected to Western analysis with ATF4 and HA antibodies as above. Actin was used as loading control.

E LNCaP cells were transfected with plasmid DNA expressing 3HA-tagged wild type STAMP2 or ferrireducase inactive mutant of STAMP2 or vector. Three days after transfection, the cytosolic (Cyt.) and nuclear fractions (Nucl.) were prepared and subjected to Western analysis with ATF and HA antibodies as above. HDAC1 and actin were used as loading controls.

**Supplementary Figure S7:**

**Figure S7. STAMP2 knockdown decreased ROS level in 22Rv1 cells.**

22Rv1 cells stably expressing srambled shRNA or shRNA targeting STAMP2 were cultured in RPMI 1460 medium containing 10% FBS. Intracellular ROS levels were then measured by CellROX reagent staining. Student’s t-test was used to analyze the significance, N=3. Error bars indicate SD. *, P = 0..0002; **, P = 0..00015.

**Supplementary Figure S8:**

**Figure S8. STAMP2 expression is decreased in PCa xenografts upon systemic treatment with nanoliposomal STAMP2 siRNAs..**

Tumor xenografts of LNCaP (A) or of VCaP cells (B) were established as described in Figure 10. In the end of the experiment, tumors (n = 4) from mice injected with empty nanoliposomes, or those containing either control or STAMP2-specific siRNA, were harvested, RNA was isolated, and qRT-PCR was performed to determine STAMP2 levels. Student’s t-test was used to analyze the significance. Error bars indicate SEM; *P < 0.05; **P < 0.05.
