# Supplementary figures and images for: STAMP2 increases oxidative stress and is critical for prostate cancer
Source: EMBO Mol Med. 2015 Feb 13;7(3):315–31. doi: 10.15252/emmm.201404181 (PMC4364948; doi:10.15252/emmm.201404181)

Supplementary Figure S3B

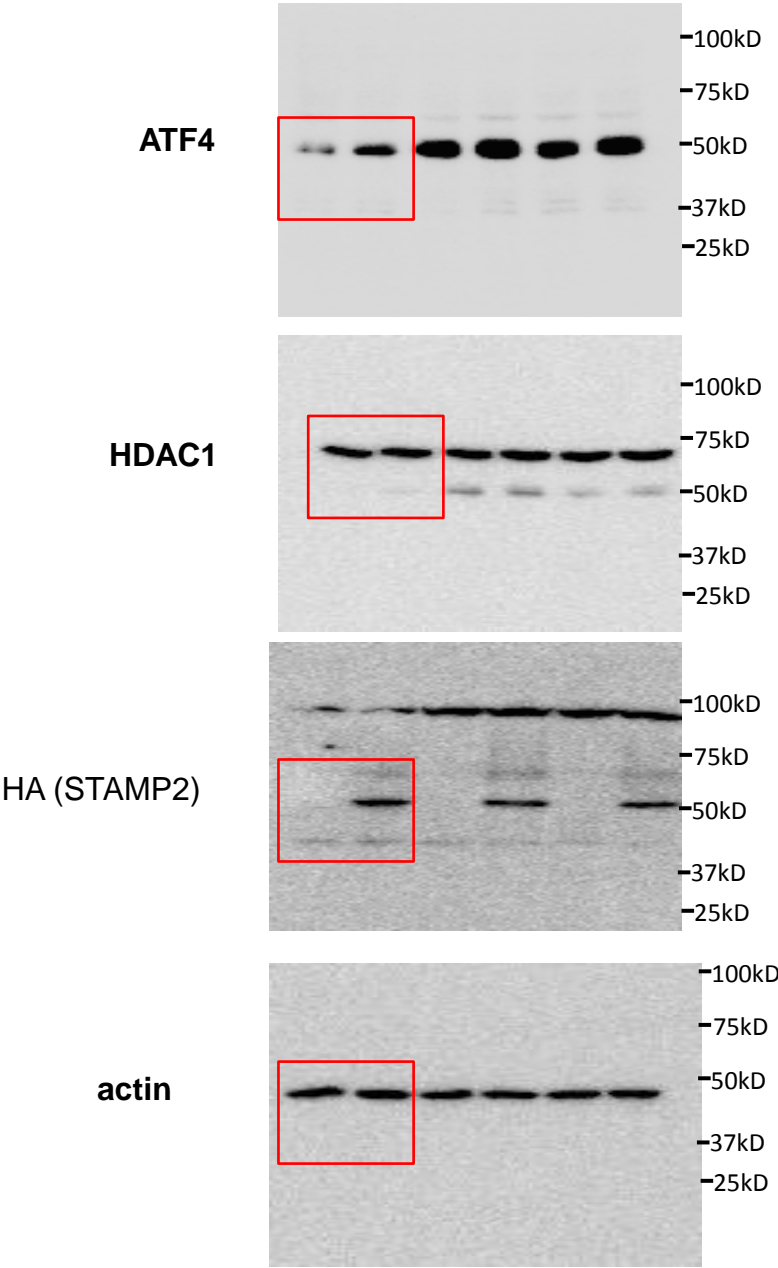

Supplement: Supplementary file 2 [file emmm0007-0315-sd2.pdf]

Figure 2A

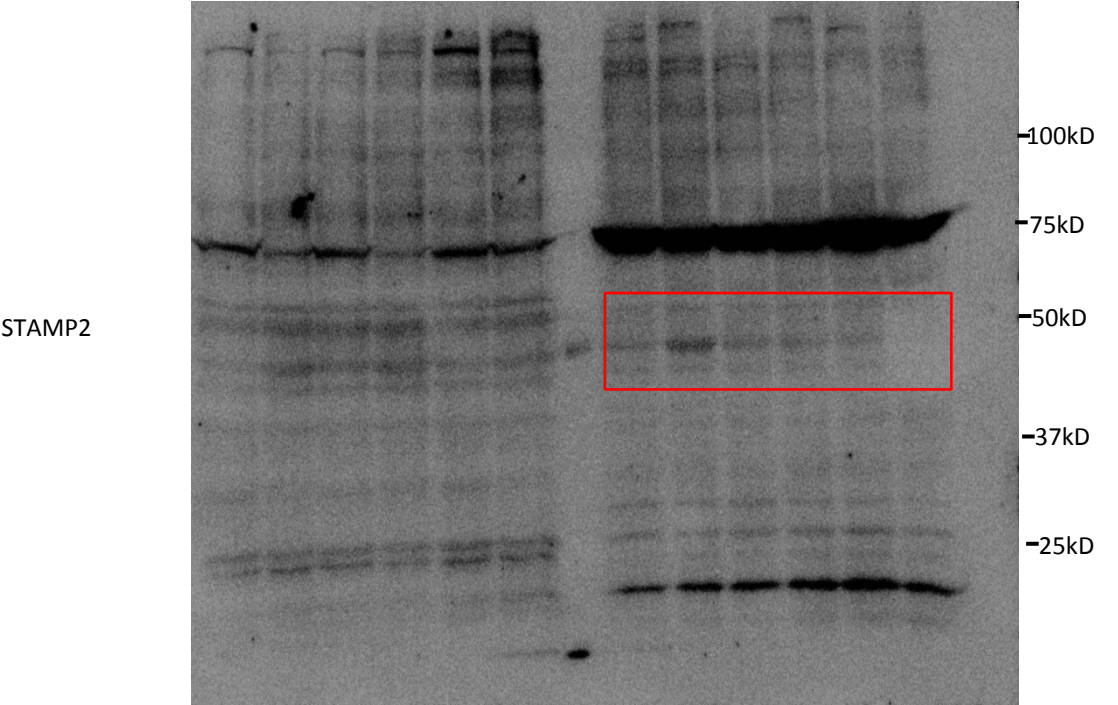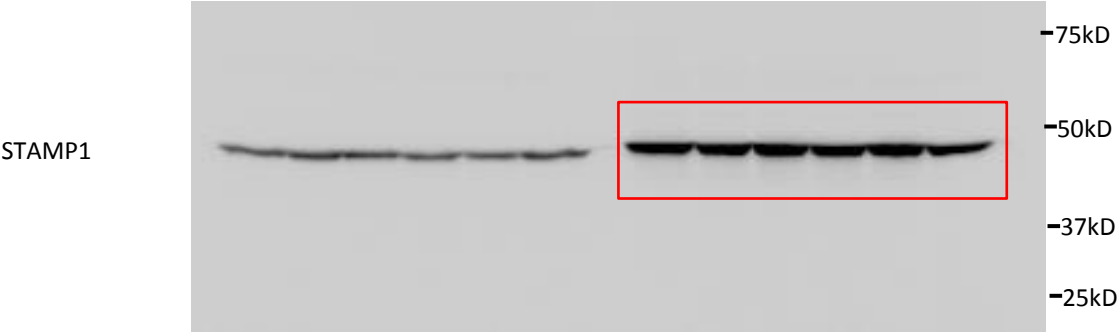

Figure 2D

Anti-STAMP2

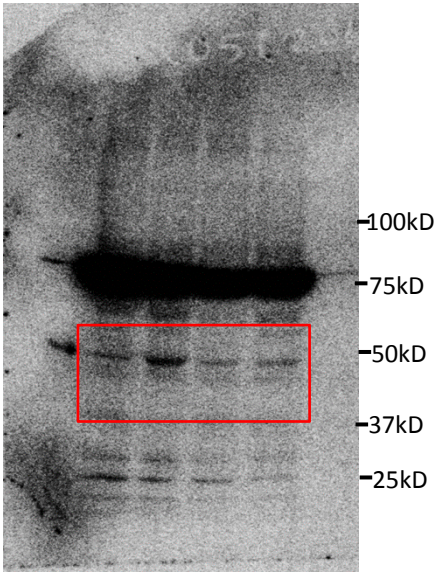

Anti-STAMP1

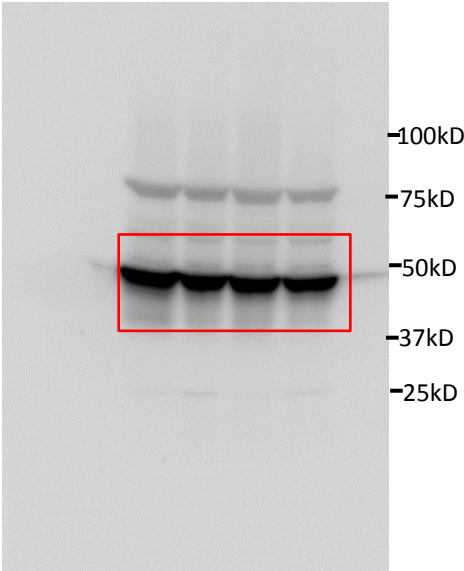

Supplement: Supplementary file 5 [file emmm0007-0315-sd5.pdf]

Figure 3C

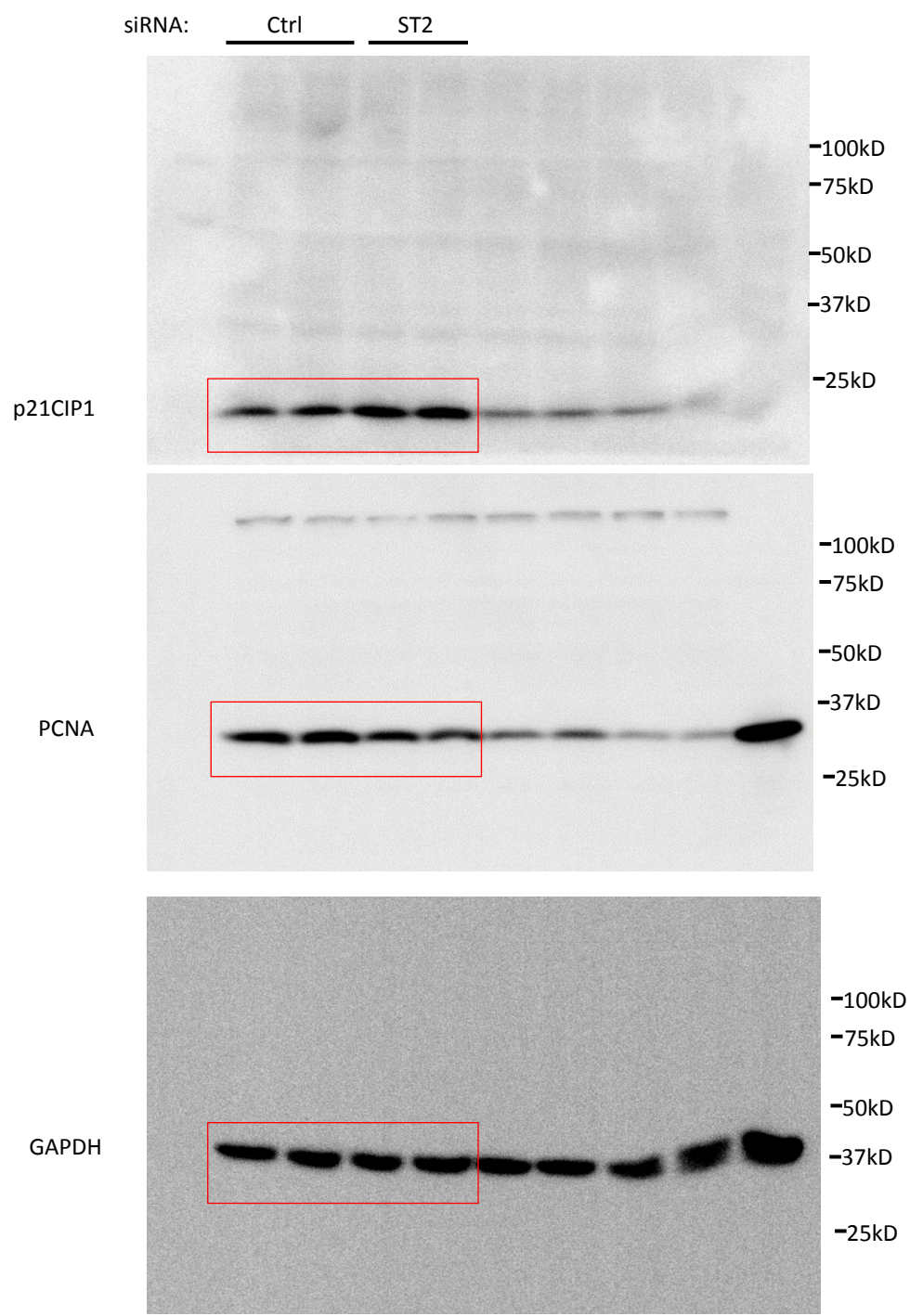

Figure 3F

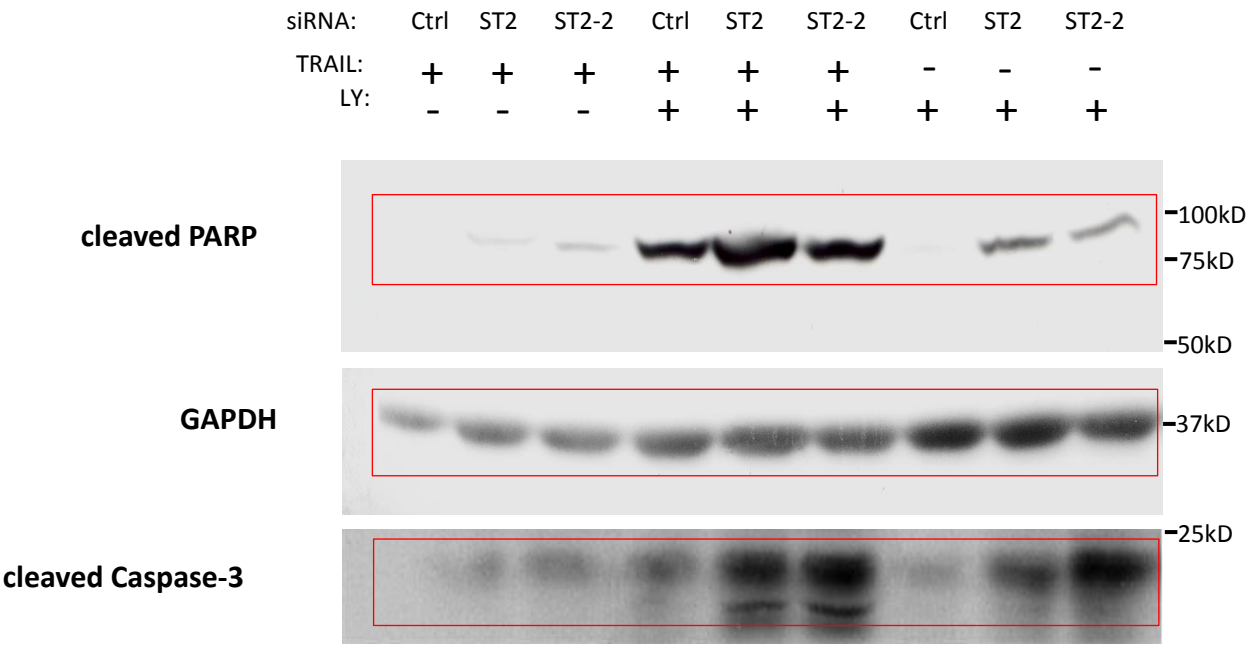

Figure 3G

cleaved Caspase-3

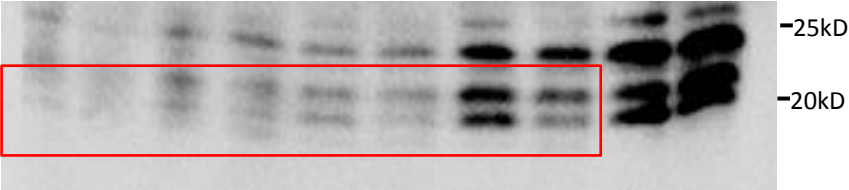

cleaved PARP

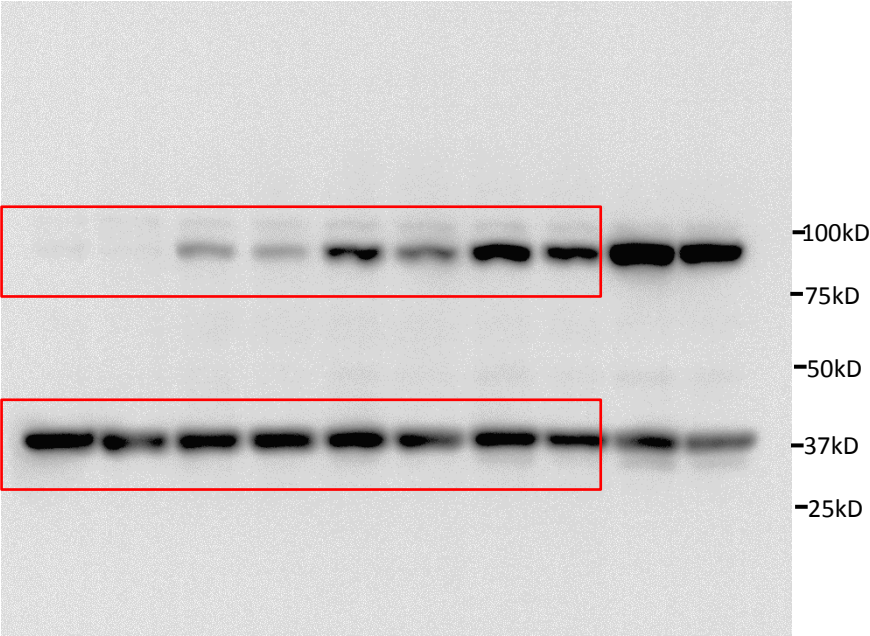

GAPDH

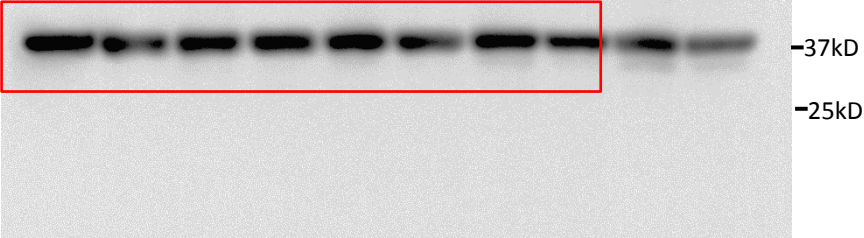

STAMP2

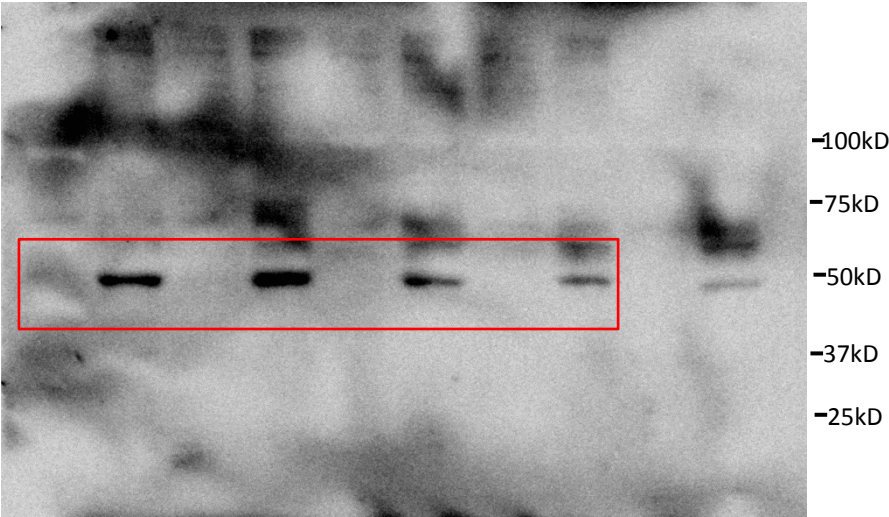

Supplement: Supplementary file 6 [file emmm0007-0315-sd6.pdf]

Figure 6E

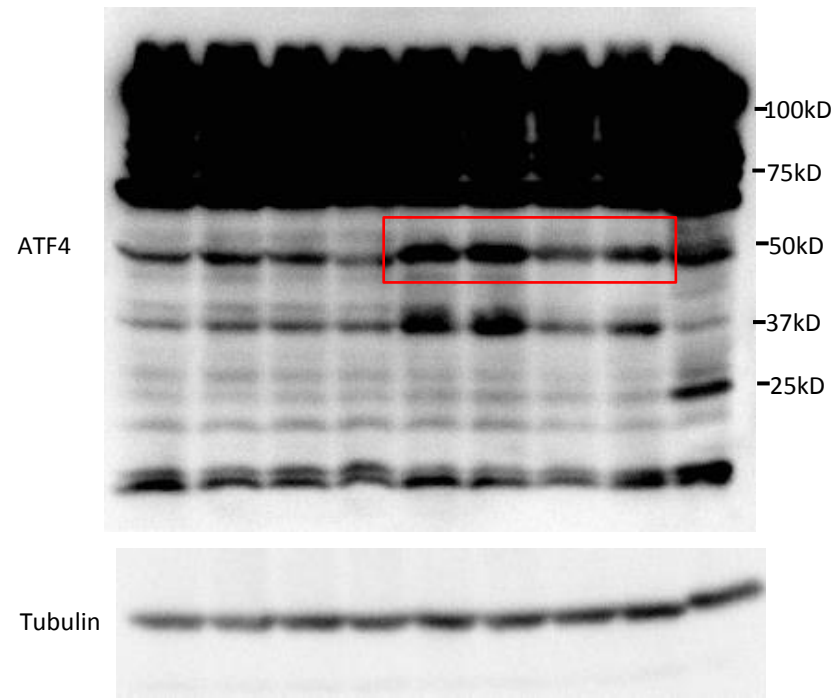

Figure 6C

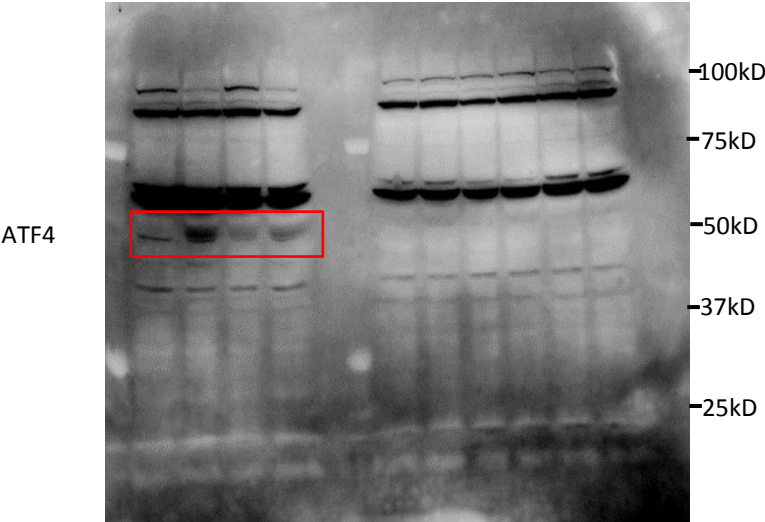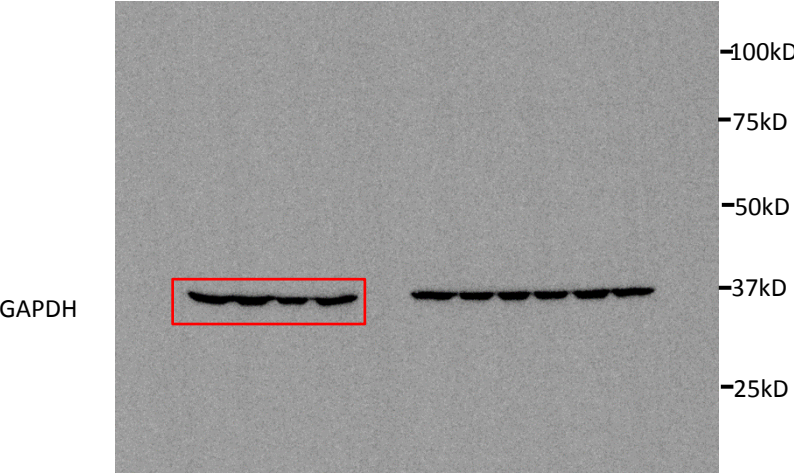

Figure 6D

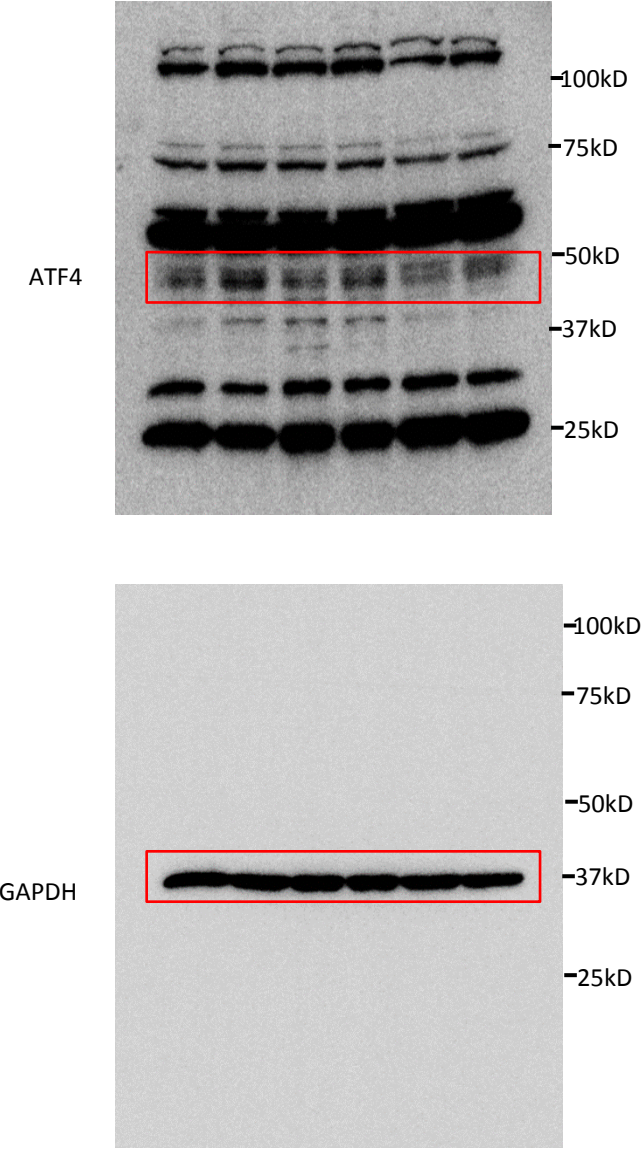

Figure 6F

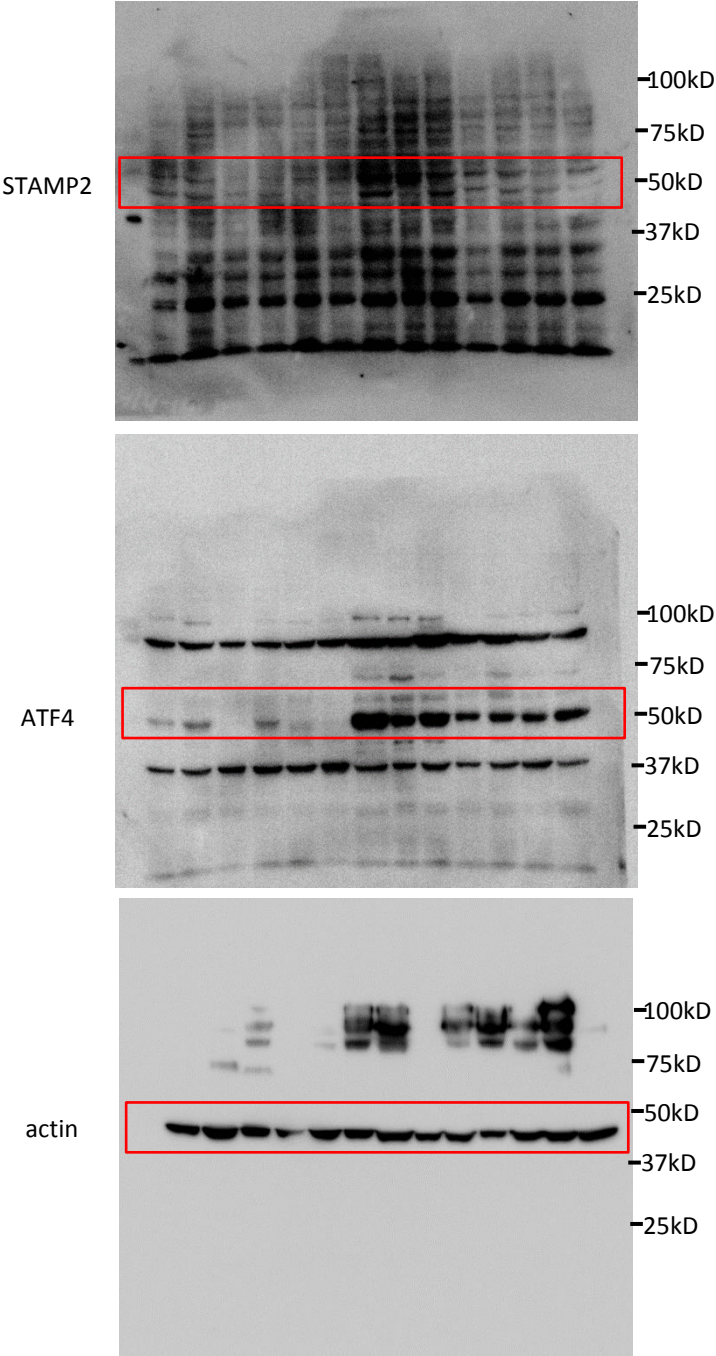

Supplement: Supplementary file 7 [file emmm0007-0315-sd7.pdf]

Figure 7A

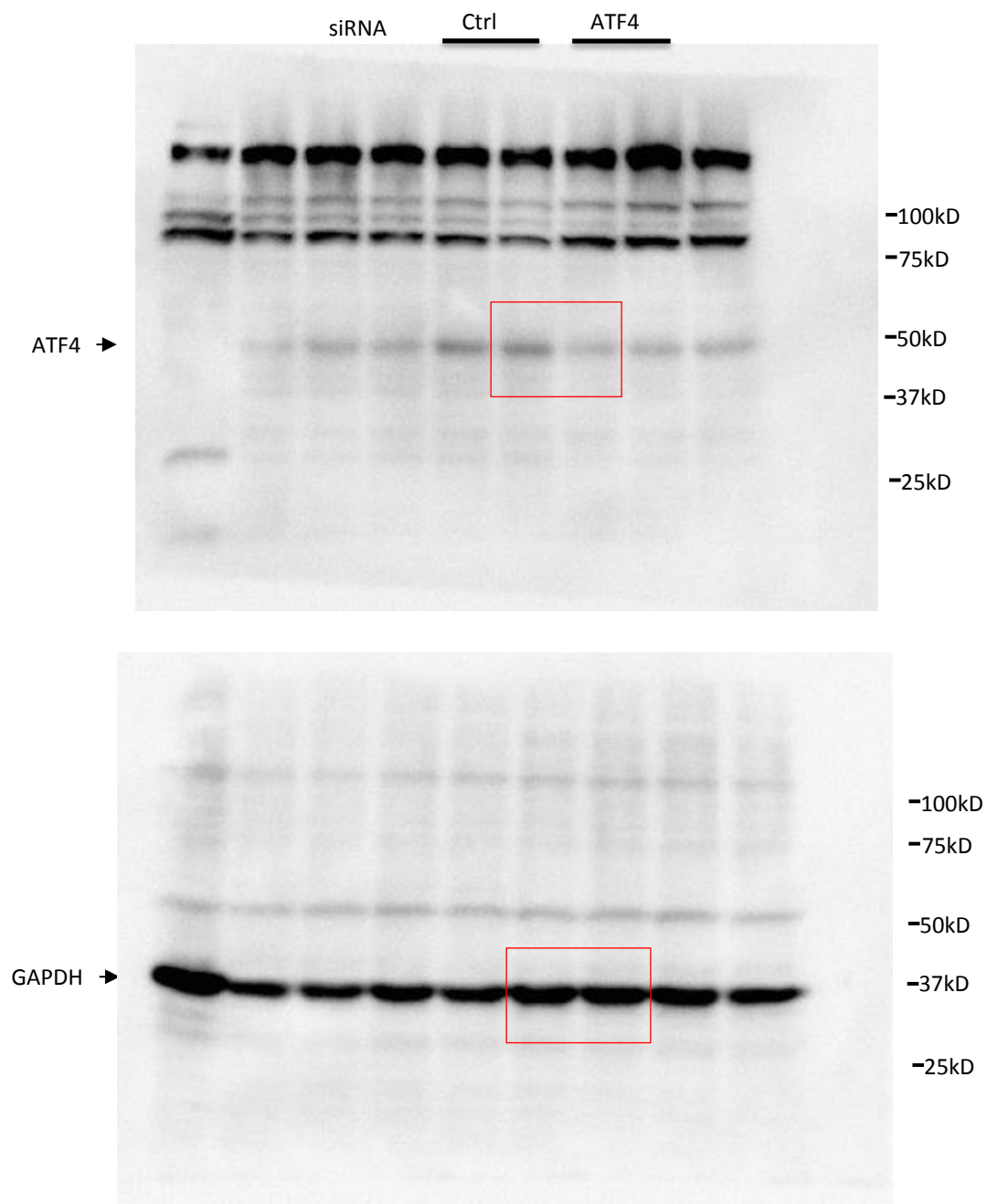

Figure 7D

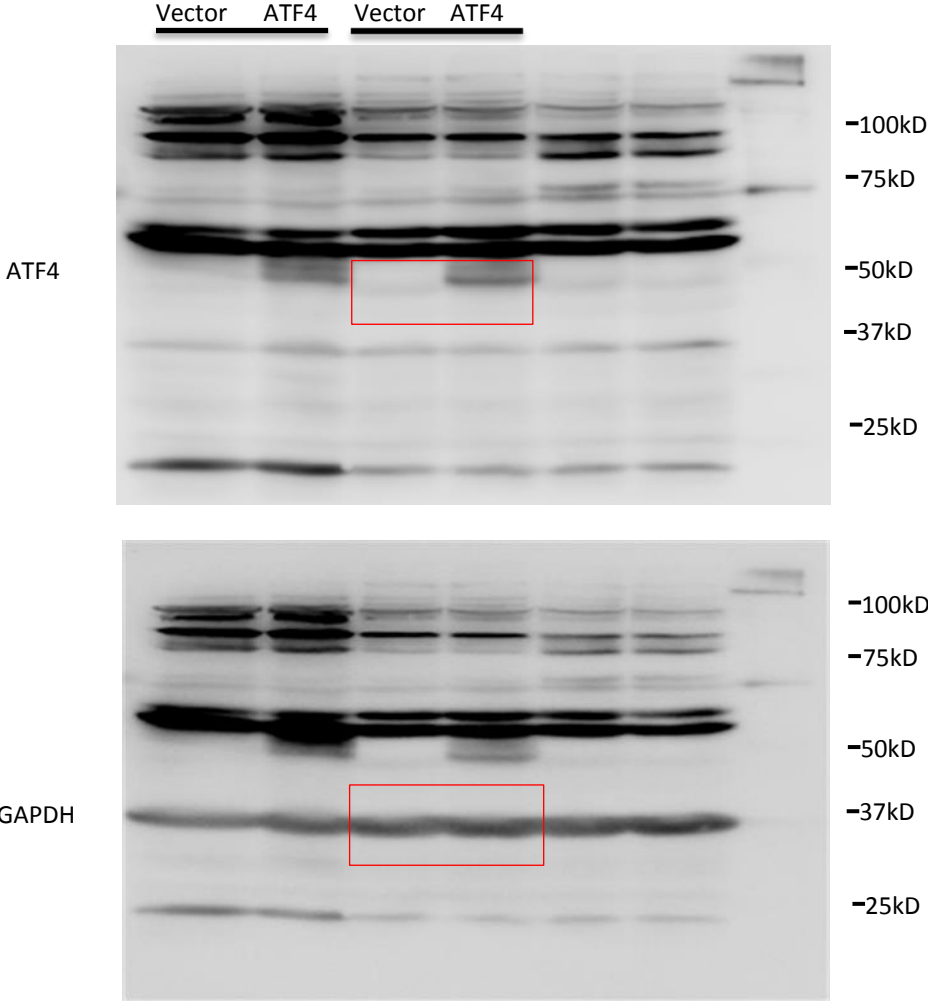

Figure 7G

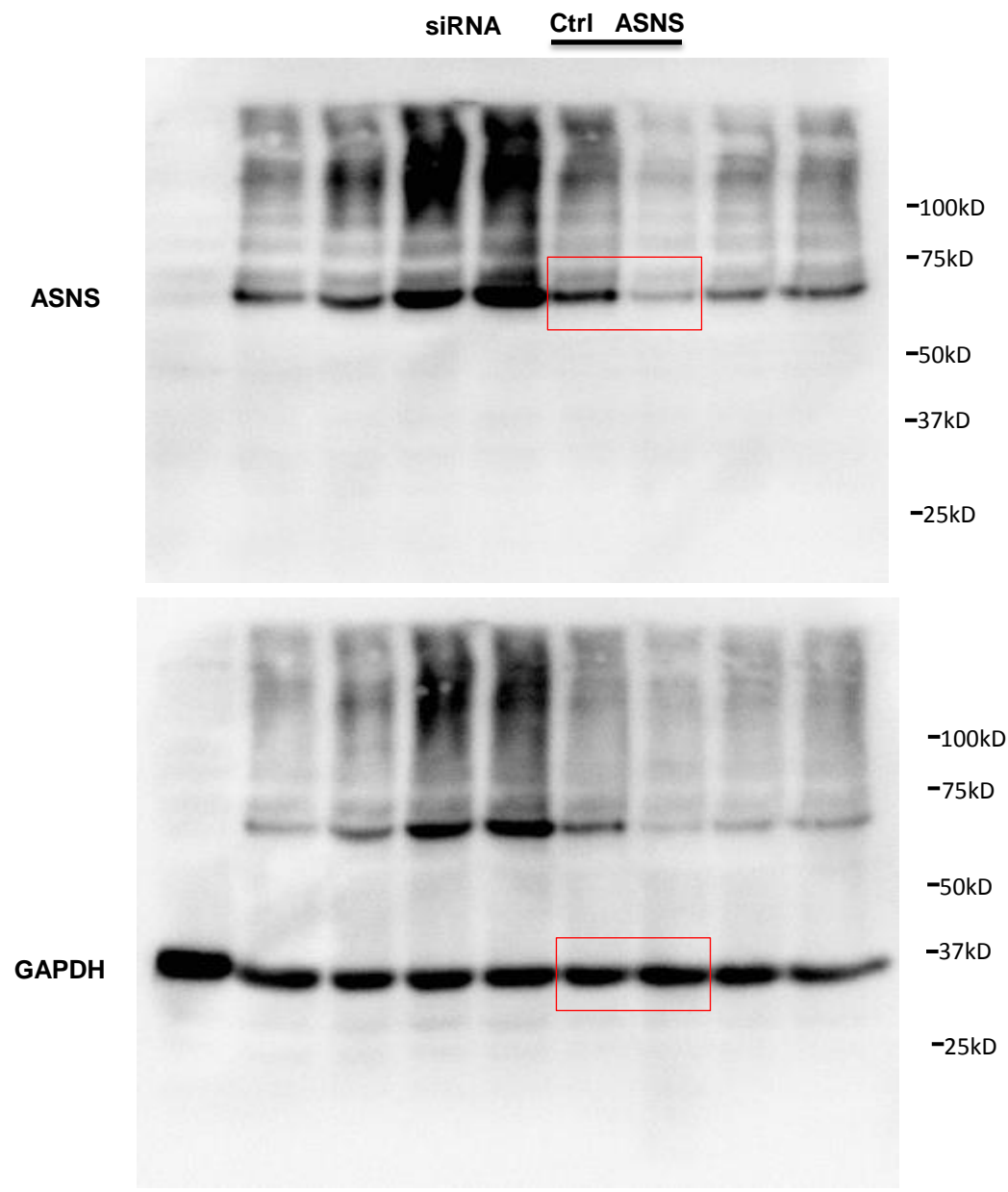

Supplement: Supplementary file 8 [file emmm0007-0315-sd8.pdf]

Figure 8A

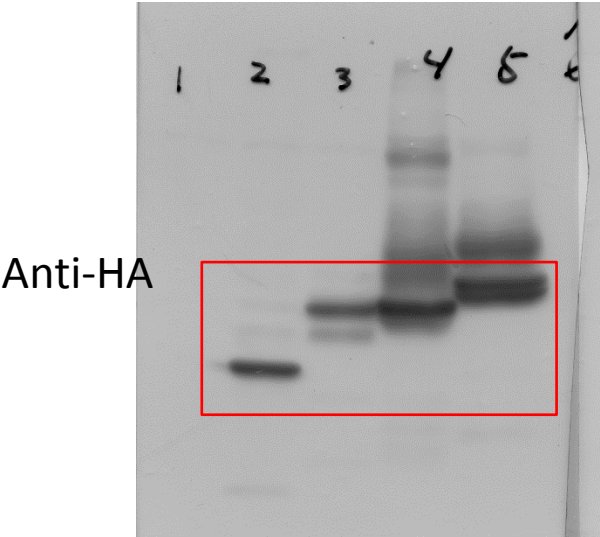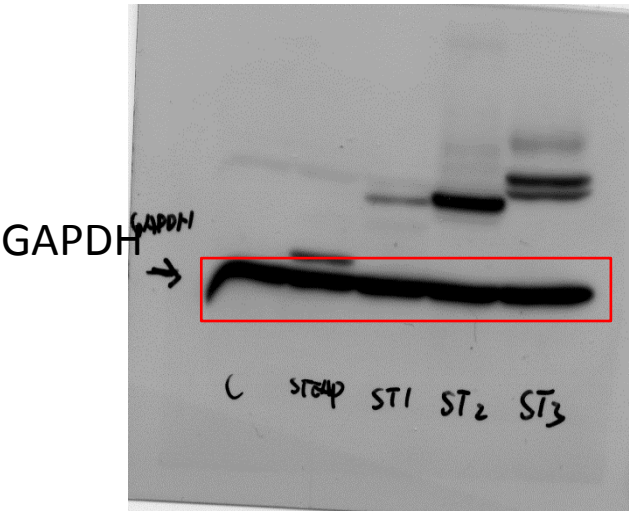

Figure 8C

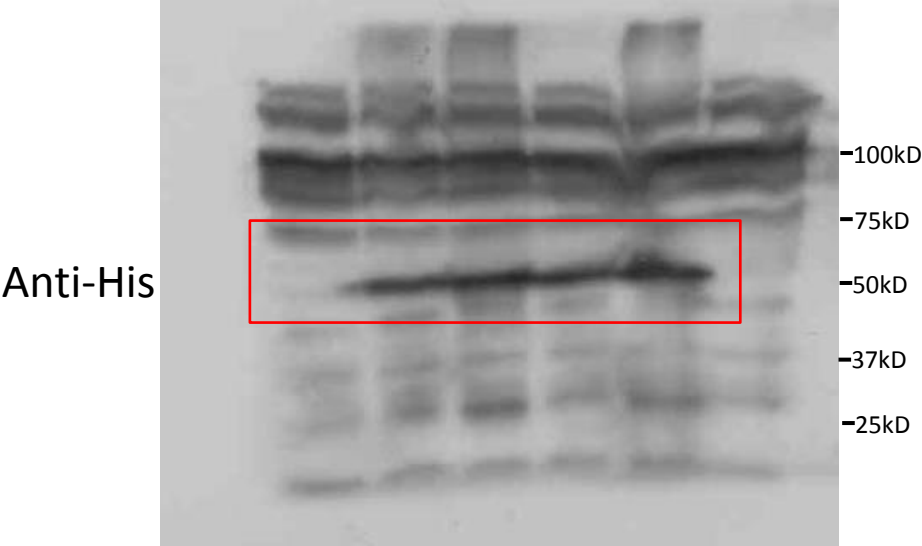

Supplement: Supplementary file 9 [file emmm0007-0315-sd9.pdf]
