# Supplementary material for: STAMP2 increases oxidative stress and is critical for prostate cancer
Source: EMBO Mol Med. 2015 Feb 13;7(3):315–31. doi: 10.15252/emmm.201404181 (PMC4364948; doi:10.15252/emmm.201404181)

Supplementary Figure S6B

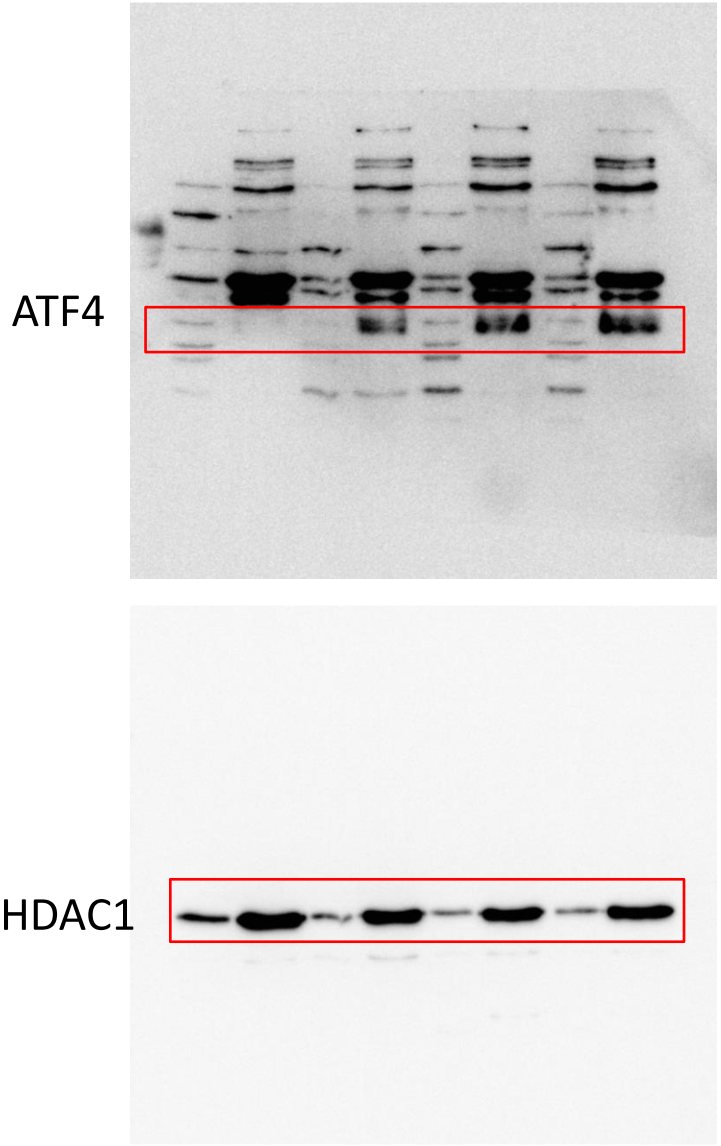

Supplementary Figure S6C

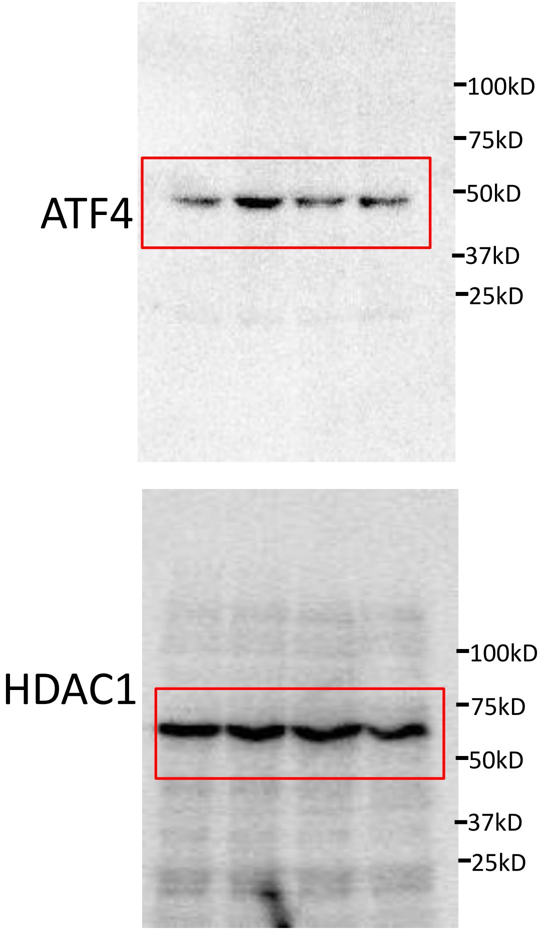

Supplementary Figure S6D

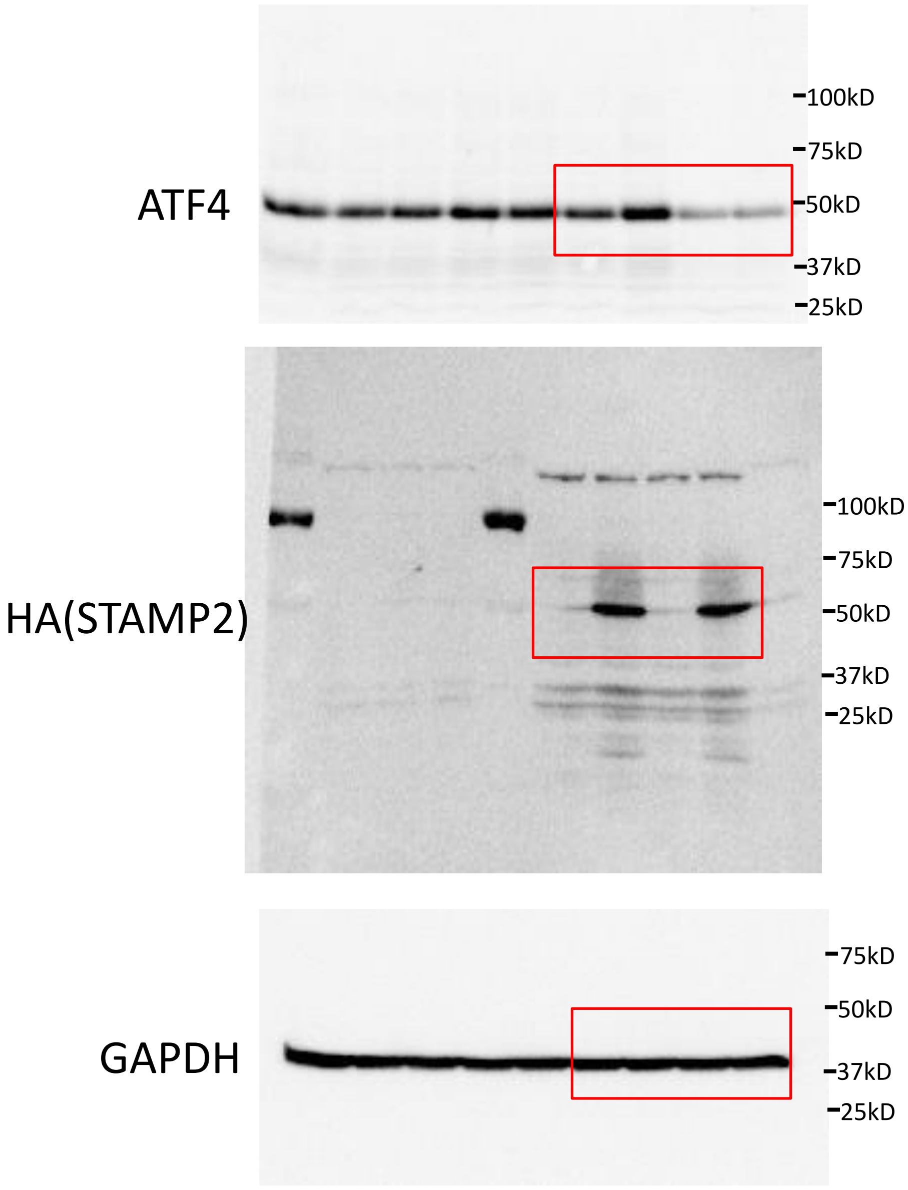

Supplementary Figure S6E

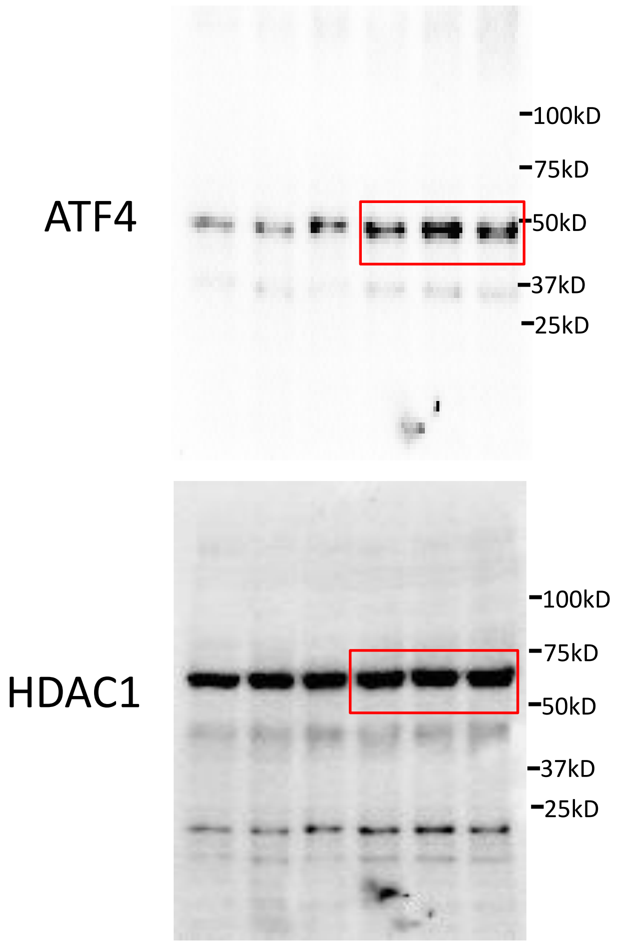

Supplement: Supplementary file 3 [file emmm0007-0315-sd3.pdf]
