# Supplementary material for: STAMP2 increases oxidative stress and is critical for prostate cancer
Source: EMBO Mol Med. 2015 Feb 13;7(3):315–31. doi: 10.15252/emmm.201404181 (PMC4364948; doi:10.15252/emmm.201404181)
Supplement: Supplementary file 4 [file emmm0007-0315-sd4.pdf]

EMBO
Molecular Medicine

STAMP2 increases oxidative stress and is critical for prostate cancer

Yang Jin, Ling Wang, Su Qu, Xia Sheng, Alexandr Kristian, Gunhild M. Mælandsmo, Nora Pällmann, Erkan Yuca, Ibrahim Tekedereli, Kivanc Gorgulu, Neslihan Alpay, Anil Sood, Gabriel Lopez-Berestein, Ladan Fazli, Paul Rennie, Bjørn Risberg, Håkon Wæhre, Håvard E. Danielsen, Bulent Ozpolat and Fahri Saatcioglu

Corresponding author: Fahri Saatcioglu, University of Oslo

Review timeline:

Submission date:	15 April 2014
Editorial Decision:	19 May 2014
Revision received:	14 October 2014
Editorial Decision:	31 October 2014
Revision received:	17 December 2014
Editorial Decision:	08 January 2015
Revision received:	14 January 2015
Accepted:	19 January 2015

Transaction Report:

(Note: With the exception of the correction of typographical or spelling errors that could be a source of ambiguity, letters and reports are not edited. The original formatting of letters and referee reports may not be reflected in this compilation.)

Editor: Roberto Buccione

1st Editorial Decision 19 May 2014

Thank you for the submission of your manuscript to EMBO Molecular Medicine. We have now heard back from the three Reviewers whom we asked to evaluate your manuscript.

You will see that the three Reviewers express several, partially overlapping concerns that prevent us from considering publication at this time. My overall impression is that all Reviewers express dissatisfaction (to varying degrees) with the experimental support provided. This includes lack of controls, need for further experimentation and over-interpretation. I would like to just mention a few specific points without dwelling into much detail, as the evaluations are detailed and self-explanatory.

Reviewer 1 would like to understand the rationale for singling out ATF4 as a STAMP2 target and in general would like to see a comparative analysis of other known datasets. S/he would also like more convincing evidence for the effect of STAMP2 knock-down on tumour growth and suggests alternative experimental approach based on xenografts with STAMP2 knock-down prostate cancer cells. This Reviewer lists other very important experimental shortcomings and requests for clarification that require your action

Reviewer 2 is especially concerned about the lack of appropriate controls and the unresolved conflicts with the existing knowledge (similarly to Reviewer 1). This Reviewer, while understanding the rationale for using LNCaP cells, also challenges their suitability based on (the

lack of) STAMP2 expression. S/he also disagrees with the use of DPI as a specific ferrireductase inhibitor and notes that some conclusions were not fully supported by the data.

Reviewer 3 also notes that crucial controls are missing and would suggest the use of an additional AR+ cell line. S/he also mentions that the androgen-independent setting should also be explored based on the patient data. This Reviewer also lists other very important issues that require your action

Considered all the above, while publication of the paper cannot be considered at this stage, we would be prepared to consider a substantially revised submission, with the understanding that the Reviewers' concerns must be fully addressed with additional experimental data where appropriate and that acceptance of the manuscript will entail a second round of review.

Since the required revision in this case appears to require a significant amount of time, additional work and experimentation and might be technically challenging, I would understand if you chose to rather seek publication elsewhere at this stage. Should you do so, we would welcome a message to this effect.

Please note that it is EMBO Molecular Medicine policy to allow a single round of revision only and that, therefore, acceptance or rejection of the manuscript will depend on the completeness of your responses included in the next, final version of the manuscript.

As you know, EMBO Molecular Medicine has a "scooping protection" policy, whereby similar findings that are published by others during review or revision are not a criterion for rejection. However, I do ask you to get in touch with us after three months if you have not completed your revision, to update us on the status. Please also contact us as soon as possible if similar work is published elsewhere.

I look forward to seeing a revised form of your manuscript as soon as possible.

***** Reviewer's comments *****

Referee #1 (Remarks):

Jin et al. studied STAMP2, an androgen-regulated protein that is overexpressed in human prostate cancer. They propose a pathway in which AR signaling upregulates STAMP2, which - through its ferrireductase activity - induces ROS and NADPH depletion, followed by activation of the ATF4 transcription factor and induction of a survival program. Overall, there is enough data to suggest this pathway, although not all pieces are solid. STAMP2 certainly seems important for the proliferation of prostate cancer cells (as thousands of other genes are).

The following points need to be addressed.

1. The gene expression data used to single out ATF4 as a STAMP2 downstream target should be shown. Is ATF4 the only gene that correlates with STAMP2 status even though there are many oxidative stress-induced transcription factors (AP1, NRF2, ATF2...)? On which basis was it chosen? It is also not clear how the data in Fig. 4C was generated. It seems the authors manually picked only those data that showed high or low STAMP2 expression (although this is not entirely clear from the short methods description). Is there a correlation between STAMP2 and ATF4 in the entire Taylor dataset? What about other published prostate cancer array datasets?
2. In Fig. 5F, it should at least be shown that there is a correlation between STAMP2 expression levels and ferrireductase activity. How can one exclude that the activity measured does not stem from another cellular reductase?
3. In the experiments shown in Fig. 5F and H, controls are missing to rule out the possibility that the effects seen are due to doxycycline rather than STAMP2 overexpression.
4. The effect on tumor growth of liposome-mediated knockdown of STAMP2 is hard to interpret, because the knockdown appears to be systemic. So it is unclear whether this is an effect on the tumor or on the host animal. Is STAMP2 actually decreased in the tumors? Prostate cancer cells with stable knockdown of STAMP2 should be constructed and their growth measured in a xenograft experiment relative to a vector only control.

5. The final model is not proven. The model predicts that androgen-mediated STAMP2-dependent ROS formation activates ATF4. It is shown that STAMP2 induces ROS and ATF4, but it remains unclear whether ATF4 induction is mediated by ROS. Do antioxidants inhibit ATF4 induction in response to STAMP2 overexpression (or indeed in response to androgen signaling)?

6. All immunoblots need size standards, and all error bars need to be defined (SD, SEM, CI?) and the number of replicates stated in the figure legends. The catalog numbers and dilutions of all antibodies should be stated.

7. The methods need to be described in more detail (e.g. ferrireductase assay, statistics, STAMP2-ATF4 correlation analysis etc...).

Referee #2 (Remarks):

The authors of this manuscript hypothesize that "STAMP2 is a critical survival factor for PCa cells in vitro and in vivo, and that it activates oxidative stress-induced ATF4 signaling through ROS generated by its iron reductase activity." While the authors make some interesting observations in this manuscript, assessing the experimental support for their conclusions is significantly challenged by several factors, including: incomplete description of methods, lack of proper controls, failure to include relevant data in figures and lack of explanations for results that are at odds with previously published reports (including those cited by the authors). Taken together, these problems indicate the manuscript should not be considered for publication as written, and specific examples supporting this assessment are provided below.

1. The scored immunohistochemical staining of STAMP2 in microarrayed human PCa tumors appears to correlate with the mRNA expression levels originally reported in their 2005 paper. However, the provided example of STAMP2 expression in benign prostate appears to have substantial expression in the luminal cells of the prostatic epithelium in what appears to be a membranous pattern (although the image quality provided to this reviewer was low), whereas all 27 of the benign samples were given a score of 0 in their membrane staining. No explanation of this discrepancy was provided.

2. Since STAMP2 expression is an androgen-regulated, the use of LNCaP cells as a model system for determining the effects of STAMP2 expression on prostate cancer aggressiveness is warranted since it is one of the few PCa cell lines that express the androgen receptor. However, endogenous STAMP2 protein expression in LNCaP was not demonstrated by any immunological means. Only when STAMP2 was ectopically expressed was the expression of STAMP2 shown at the protein level. This makes it very difficult to assess the potential contribution of STAMP2 protein expression on the phenotypic measurements provided. In addition, no description of the methodology for quantitative PCR used to support for the effectiveness of STAMP2 siRNA was provided.

3. The authors make the interesting observation that the expression of ATF4 is dramatically reduced in LNCaP cells treated with the androgenic compound R1881 specifically when STAMP2 expression is inhibited by siRNA and that ATF4 expression correlated with the expression of STAMP2 in PCa as determined using data obtained from cBio (MSKCC). However, no description of how these data were analyzed was provided.

4. This reviewer questions whether the 3-5% reduction in relative ROS levels in response to decreased of STAMP2 expression in LNCaP cells (Fig. 6E) is sufficient to account for the biological effects being attributed to STAMP2 ferrireductase activity.

5. The use of diphenyleneiodonium sulfate (DPI) as a specific ferrireductase inhibitor (as the authors claim) is called into question. http://www.jbc.org/content/279/46/47726.full

6. The hypothesis that reduction of STAMP2 expression by siRNA would directly lead to tumor reduction (as shown in Fig. 7) is at odds with the authors' own data that was summarized with the statement, "There were no significant changes in basal levels of apoptosis upon STAMP2 knockdown (data not shown)."

Referee #3 (Comments on Novelty/Model System):

While several instances of attempts to generalize the findings can be seen in the figures, it seems odd to compare results related to an AR/androgen-responsive gene in LNCaP (AR+) and DU-145 (AR-) cells, and there should be an effort made to reproduce LNCaP results in another AR+ cell model of ADPC. Culture conditions for DU-145 are not provided making it difficult for the reader to compare these findings to those in LNCaP.

Related to the above Comment , given that patients with recurrent disease following hormone therapy exhibit restored STAMP2 expression, it seems germane to this study to explore androgen independent STAMP2 expression in an appropriate cell line model.

Referee #3 (Remarks):

This manuscript describes a very interesting story in which the investigators revealed a role for STAMP2 as a ferrireductase responsible for the accumulation of ROS in prostate cancer cells, leading to enhanced cell proliferation via ATF4 signaling. This study nicely links several previous observations, namely that deregulated androgen signaling can stimulate ROS production and prostate cancer development is associated with oxidative stress. The authors find that the protein expression of STAMP2, previously described as an androgen-regulated gene, correlates with Gleason score and shows responsiveness to neoadjuvant hormone therapy, even rising after the development of treatment resistance. Many in vitro and in vivo analyses then demonstrate that STAMP2 expression is required for LNCaP cell growth, LNCaP xenograft tumor formation, G1 progression, and survival. Gene expression analysis following STAMP2 silencing revealed ATF4 to be significantly down-regulated, suggesting this important transcription factor is downstream of STAMP2 signaling. ATF4 too was shown to be required for LNCaP cell growth. After establishing the ferrireductase activity of human STAMP2 in 293T cells, the authors show that STAMP2 expression and activity leads to ROS accumulation and a concordant loss of NADPH in prostate cancer cells. Finally, the authors demonstrate the therapeutic value of targeting STAMP2 in prostate cancer by delivering nanoliposomal siSTAMP2 to mice injected with LNCaP or VCaP xenografts.

While I certainly like the story told by this work, I don't feel that proper/rigorous controls have been included. Thus, I would like to see a several issues addressed, and I hope the authors will resubmit this work for future review.

Comments:

1) In general, given the androgen-responsiveness of STAMP2, I believe that standard/accepted cell culture methods (10% CSS +/- DHT/R1881) should be employed for all assays and that the vast majority of experiments need to include controls for hormone depleted conditions.

2) While several instances of attempts to generalize the findings can be seen in the figures, it seems odd to compare results related to an AR/androgen-responsive gene in LNCaP (AR+) and DU-145 (AR-) cells, and there should be an effort made to reproduce LNCaP results in another AR+ cell model of ADPC. Culture conditions for DU-145 are not provided making it difficult for the reader to compare these findings to those in LNCaP.

3) Related to Comment 2, given that patients with recurrent disease following hormone therapy exhibit restored STAMP2 expression, it seems germane to this study to explore androgen independent STAMP2 expression in an appropriate cell line model.

4) Figure 4: What is the statistical correlation between ATF4 and STAMP2 expression in the MSKCC data?

5) Figure 4: Could you explore the response of some ATF4 target genes (perhaps relevant to prostate cancer, cell survival, etc.) to modulation of STAMP2 expression and activity?

6) Figure 6E: The text says this panel is data for DU-145, but the figure legend describes them as LNCaP cell data.

7) Figure 7: Did these experiments reach statistical significance? The figure legend describes p-values, but no indication of significance can be found.

1st Revision - authors' response 14 October 2014

Referee #1:

1. The gene expression data used to single out ATF4 as a STAMP2 downstream target should be shown. Is ATF4 the only gene that correlates with STAMP2 status even though there are many oxidative stress-induced transcription factors (AP1, NRF2, ATF2...)? On which basis was it chosen? It is also not clear how the data in Fig. 4C was generated. It seems the authors manually picked only those data that showed high or low STAMP2 expression (although this is not entirely clear from the short methods description). Is there a correlation between STAMP2 and ATF4 in the entire Taylor dataset? What about other published prostate cancer array datasets?

To determine signaling pathways that may be affected by STAMP2, we carried out a global gene expression profiling in LNCaP cells in which STAMP2 was knocked down compared with wild type LNCaP cells. As shown in Supplementary Figure S2A, we found that ATF4, as well as several ATF4 target genes, were on the list of genes whose expression was most strongly affected. There were no significant alterations in the expression of other stress-induced transcription factors. We thus hypothesized that ATF4 signaling may be associated with STAMP2 function and investigated possible correlation in clinical human PCa cohorts. This included analysis of normalized gene expression profiles of PCa samples (n=150) from a MSKCC PCa cohort (source data from http://cbio.mskcc.org/cancergenomics/prostate/data/). The samples were aligned based on the expression level of STAMP2. In this case, 30 samples with lower STAMP2 expression was set as STAMP2_low, while 30 samples with higher STAMP2 expression was set as STAMP2_high. The correlation between STAMP2 and genes of interest was analyzed by using GSEA program from Broad Institute (www.broadinstitute.org/gsea). In this revised manuscript, instead of analyzing the correlation of STAMP2 expression and only that of ATF4, we designed a gene set including ATF4 and two ATF4 targets, ASNS and SLC7A11 and compared their expression with that of STAMP2. The updated data are now presented as Figure 6G. The significance of the correlation between ATF4 signaling and STAMP2 in the entire Taylor dataset was calculated using Pearson correlation coefficient analysis. The data shows a positive correlation ($R=0.4015$) and is statistically significant ($p<0.00001$). Similar results were obtained in two independent PCa gene expression datasets as shown in Supplementary Figures 2B and C. Altogether, we feel that these data robustly link an association between STAMP2 and ATF-4 signaling in human PCa specimens.

2. In Fig. 5F, it should at least be shown that there is a correlation between STAMP2 expression levels and ferrireductase activity. How can one exclude that the activity measured does not stem from another cellular reductase?

The data requested by the reviewer are now presented in Figure 8 of the revised manuscript. We have now included the control cell line that harbors an empty vector in these experiments. The dose-dependent induction of STAMP2 expression is shown in Figure 8E; in parallel, a side-by-side measurement of ferrireductase activity in the control and STAMP2 cell lines was carried out as shown in Figure 8F. Whereas there was no induction of ferrireductase activity by doxycycline in control cells, this activity was dramatically, and in a dose dependent manner, induced in STAMP2 cells showing that the ferrireductase activity is due to STAMP2 expression. Consistently, inactivation of STAMP2 ferrireductase activity by mutagenesis at the flavin or heme binding motifs blocked ferrireductase activity further confirming these findings (Figure 5D).

3. In the experiments shown in Fig. 5F and H, controls are missing to rule out the possibility that the effects seen are due to doxycycline rather than STAMP2 overexpression.

These have now been provided. Please see the above response in pt 2.

4. The effect on tumor growth of liposome-mediated knockdown of STAMP2 is hard to interpret, because the knockdown appears to be systemic. So it is unclear whether this is an effect on the tumor or on the host animal. Is STAMP2 actually decreased in the tumors? Prostate cancer cells

© EMBO

with stable knockdown of STAMP2 should be constructed and their growth measured in a xenograft experiment relative to a vector only control.

It is known that the systemically administered nanoliposomal siRNAs are preferentially taken up by tumor cells which exhibit the enhanced permeation and retention (EPR) effect that enables nanomaterials to accumulate and be retained in the tumor (for a review, see Schroeder et al., Nat Rev. Cancer 2012, v12: 39-50). In addition, the siRNA targeting STAMP2 is specific for the human mRNA and would not affect the mouse Stamp2 in the host animal tissues. Regarding the xenograft experiment suggested, we have already presented these data in Figure 2H of the original submission. Furthermore, we have now extended these results to 22Rv1 xenografts representing CRPC where we obtained consistent data (Figure 5).

5. The final model is not proven. The model predicts that androgen-mediated STAMP2-dependent ROS formation activates ATF4. It is shown that STAMP2 induces ROS and ATF4, but it remains unclear whether ATF4 induction is mediated by ROS. Do antioxidants inhibit ATF4 induction in response to STAMP2 overexpression (or indeed in response to androgen signaling)?

To show that ATF4 induction is mediated by ROS in PCa cells, we have used menadione which has been shown to increase ROS in other cell types (e.g. Perez Soler et al., Clin Cancer Res. 2011, v17: 6766-6777; Chowdury et al., Apoptosis. 2009, v14:108-123). As shown in Supplementary Figure S3, menadione treatment significantly increased ROS levels in LNCaP cells and induced ATF4 expression.

6. All immunoblots need size standards, and all error bars need to be defined (SD, SEM, CI?) and the number of replicates stated in the figure legends. The catalog numbers and dilutions of all antibodies should be stated.

We have now provided size standards for all Western blots, as well as detailed information on error bars and additional details of the experiments in figure legends. The catalog numbers and dilutions of all antibodies are now provided in the Methods.

7. The methods need to be described in more detail (e.g. ferrireductase assay, statistics, STAMP2-ATF4 correlation analysis etc...).

We have now updated the Methods section with detailed description of the procedures.

Referee #2:

1. The scored immunohistochemical staining of STAMP2 in microarrayed human PCa tumors appears to correlate with the mRNA expression levels originally reported in their 2005 paper. However, the provided example of STAMP2 expression in benign prostate appears to have substantial expression in the luminal cells of the prostatic epithelium in what appears to be a membranous pattern (although the image quality provided to this reviewer was low), whereas all 27 of the benign samples were given a score of 0 in their membrane staining. No explanation of this discrepancy was provided.

A more representative picture for the benign prostate is now provided. The scoring was done such that no to very low expression was given a score of 0 which was the case in 23 out of 27 samples. This is now described more clearly in the Methods section, under 'Scoring and statistical analysis of TMAs′.

2. Since STAMP2 expression is an androgen-regulated, the use of LNCaP cells as a model system for determining the effects of STAMP2 expression on prostate cancer aggressiveness is warranted since it is one of the few PCa cell lines that express the androgen receptor. However, endogenous STAMP2 protein expression in LNCaP was not demonstrated by any immunological means. Only when STAMP2 was ectopically expressed was the expression of STAMP2 shown at the protein level. This makes it very difficult to assess the potential contribution of STAMP2 protein expression on the phenotypic measurements provided. In addition, no description of the methodology for quantitative PCR used to support for the effectiveness of STAMP2 siRNA was provided.

Since STAMP2 expression is very strongly androgen dependent, there is very low level of the STAMP2 protein in uninduced cells which is not detectable in whole cell extracts by Western analyses. We have now prepared membrane fractions, as described in Methods, and were able to detect STAMP2 basal levels in Western analyses in both transient (Figure 2A) and stable knockdown (Figure 2D) of STAMP2 in LNCaP cells.

3. The authors make the interesting observation that the expression of ATF4 is dramatically reduced in LNCaP cells treated with the androgenic compound R1881 specifically when STAMP2 expression is inhibited by siRNA and that ATF4 expression correlated with the expression of STAMP2 in PCa as determined using data obtained from cBio (MSKCC). However, no description of how these data were analyzed was provided.

The details of these analyses as requested by the reviewer and further data have now been presented in the revised manuscript. Please see the response to Reviewer #1, pt. 1.

4. This reviewer questions whether the 3-5% reduction in relative ROS levels in response to decreased of STAMP2 expression in LNCaP cells (Fig. 6E) is sufficient to account for the biological effects being attributed to STAMP2 ferrireductase activity.

The reduction in ROS levels (measured by DHE staining), although limited in scope, is significant from experiments repeated several times. In the revised manuscript, we confirmed this observation using a new oxidative stress detection reagent, CellROX® Deep Red Reagent (Life Technologies). As shown in Figure 9D, there was a significant increase (~3 fold) of ROS levels in LNCaP cells in response to androgen treatment. STAMP2 knockdown significantly decreased androgen induced ROS levels (10%), while there was no significant difference in the absence of androgen, i.e. when there was no/very low STAMP2 present. We have also obtained consistent results in 22RV1 cells (Supplementary Figure S5). Our interpretation of these robust data is that there may be a delicate balance of ROS in PCa cells which could be affected by relatively small changes induced by STAMP2. Another possibility is that the assays used have high basal level of detection sensitivity ('background') in these cell lines. Taken together, we feel that the data we present are robust and show that STAMP2 regulates ROS levels in PCa cells.

5. The use of diphenyleneiodonium sulfate (DPI) as a specific ferrireductase inhibitor (as the authors claim) is called into question. http://www.jbc.org/content/279/46/47726.full

We thank the reviewer for pointing this out. We have now taken this into consideration in describing DPI in the revised manuscript. Furthermore, we confirmed the ferrireductase-dependent generation of ROS by STAMP2 in an experiment where an FAD binding mutant of STAMP2 was used which has lost this activity (Figure 9C).

6. The hypothesis that reduction of STAMP2 expression by siRNA would directly lead to tumor reduction (as shown in Fig. 7) is at odds with the authors' own data that was summarized with the statement, "There were no significant changes in basal levels of apoptosis upon STAMP2 knockdown (data not shown)."

The statement the Reviewer refers to is describing data from *in vitro* experiments (Figure 3). In contrast, the tumor reduction experiments are conducted *in vivo* (the old Figure 7, Fig. 10 in the revised manuscript). Tumor cells *in vitro* have completely different microenvironments compared with those *in vivo*. For example, it is known that tumor cells are under several different stresses (oxygen and nutrient deprivation) compared with ideal growth conditions *in vitro* monolayer culture. It is thus possible that these signals sensitize the cells to apoptosis which is then further increased upon STAMP2 knockdown. Further work is required to determine the molecular details of these differences.

Referee #3:

1. While several instances of attempts to generalize the findings can be seen in the figures, it seems odd to compare results related to an AR/androgen-responsive gene in LNCaP (AR+) and DU-145 (AR-) cells, and there should be an effort made to reproduce LNCaP results in another AR+ cell

model of ADPC. Culture conditions for DU-145 are not provided making it difficult for the reader to compare these findings to those in LNCaP.

We have now deleted the data from DU-145 cells and conducted experiments in another AR+ cell model of ADPC, VCaP cells. Consistent results were obtained as presented in Figure 2I and J.

2. Related to the above Comment, given that patients with recurrent disease following hormone therapy exhibit restored STAMP2 expression, it seems germane to this study to explore androgen independent STAMP2 expression in an appropriate cell line model.

In line with this request, we have now provided significant new data regarding the role of STAMP2 in hormone-refractory PCa. We show that STAMP2 expression is associated with the development of CRPC in both human PCa cohorts (Figure 4C) and in human androgen dependent xenograft model progressing to CRPC (Figure 4D). Furthermore, consistent with a role of STAMP2 in progression to CRPC, STAMP2 knockdown significantly inhibited, both *in vitro* and *in vivo,* the growth of AR+ but androgen-insensitive PCa cell line 22Rv1 that models CRPC (Figure 5).

3) *In general, given the androgen-responsiveness of STAMP2, I believe that standard/accepted cell culture methods (10% CSS +/- DHT/R1881) should be employed for all assays and that the vast majority of experiments need to include controls for hormone depleted conditions.*

We have now provided these data in the 10%CSS +/- R1881(Figure 2, Figure 5, Figure 6C, Figure 6D and Figure 9D).

4) While several instances of attempts to generalize the findings can be seen in the figures, it seems odd to compare results related to an AR/androgen-responsive gene in LNCaP (AR+) and DU-145 (AR-) cells, and there should be an effort made to reproduce LNCaP results in another AR+ cell model of ADPC. Culture conditions for DU-145 are not provided making it difficult for the reader to compare these findings to those in LNCaP.

Please see the response above in pt. 2.

5) Related to Comment 2, given that patients with recurrent disease following hormone therapy exhibit restored STAMP2 expression, it seems germane to this study to explore androgen independent STAMP2 expression in an appropriate cell line model.

Please see the response above in pt. 2.

6) Figure 4: What is the statistical correlation between ATF4 and STAMP2 expression in the MSKCC data?

The correlation is significant and p value (, $p<0.00001$) is now provided in the text (p8, last paragraph).

7) Figure 4: Could you explore the response of some ATF4 target genes (perhaps relevant to prostate cancer, cell survival, etc.) to modulation of STAMP2 expression and activity?

We have now updated the analysis in multiple data sets by including ATF4 target genes ASNS and SLC7A11. We obtained similar results to those of ATF4 alone (Supplementary Figure S3). In addition, we have found that knockdown of ASNS, one of the ATF4 targets, in LNCaP cells inhibited cell growth (Figure 7G and H). These data even more firmly implicate ATF4 signaling as a mediator of STAMP2 effects in PCa cells.

6) Figure 6E: The text says this panel is data for DU-145, but the figure legend describes them as LNCaP cell data.

As described above, we have now removed these data in the revised manuscript.

7) Figure 7: Did these experiments reach statistical significance? The figure legend describes p-values, but no indication of significance can be found.

Yes, the differences did reach statistical significance which is now indicated both in the figure and the legend (new Figure 10).

2nd Editorial Decision 31 October 2014

Thank you for the submission of your revised manuscript to EMBO Molecular Medicine. We have now heard back from the three Reviewers whom we asked to re-evaluate your manuscript.

You will see that while Reviewer 3 is now globally positive, Reviewers 1 and 2 still have important reservations.

An important and shared item of concern is that the claim that STAMP2-induced ATF4 expression depends on ROS remains unsupported by the experimental data. Indeed, Reviewers 1 and 2 insist on provision of more conclusive experimentation to this effect.

Reviewer 1 also mentions other items that require further action on your part including the need for improved statistical documentation and provision of controls.

Reviewer 2 also lists another item of concern, i.e. the lack of a satisfactory response on his/her initial comment that STAMP2 expression in the benign sample appeared to have membranous staining in contrast with the assigned score.

We agree that these issues are relevant and are not further-reaching with respect to the original Reviewer requests and thus we cannot offer publication at this stage.

Although it is EMBO Molecular Medicine policy to allow a single round of experimental revision only, after discussion with my colleagues, I have decided to allow you to submit a re-revised version that must include the required experimentation, which does not appear to be excessively lengthy and or/complex, and a full rebuttal.

Acceptance or rejection of the manuscript will depend on the completeness of your responses included in the next, final version of the manuscript.

Please note that EMBO Molecular Medicine now requires a complete author checklist (http://embomolmed.embopress.org/authorguide#editorial3) to be submitted with all revised manuscripts. Provision of the author checklist (also attached for your convenience) is mandatory at revision stage; The checklist is designed to enhance and standardize reporting of key information in research papers and to support reanalysis and repetition of experiments by the community. The list covers key information for figure panels and captions and focuses on statistics, the reporting of reagents, animal models and human subject-derived data, as well as guidance to optimise data accessibility.

I look forward to seeing a revised form of your manuscript as soon as possible.

***** Reviewer's comments *****

Referee #1 (Remarks):

The authors have addressed a number of my previous comments and the manuscript is improved. Improvements include a documentation of the knockdown efficiency of endogenous STAMP2 protein, the inclusion of a mutant STAMP2 protein deficient in FAD binding, and the addition of size markers to most (not all) blots. The methods description was also improved, although the ferrireductase assay is still not described, and the statistical tests are insufficiently described.
1. Fig. 4C and D is described as showing "statistically significant differences", although it was not indicated which statistical tests were done.

2. Fig. 8H: It looks like there are no cells in the NBT negative dishes shown. Some sort of counterstaining should be shown to document the presence of cells in the negative dishes.
3. The final model in Fig. 10C remains unproven. The fact that STAMP2 induces ROS and that a chemical ROS inducer upregulates ATF4 does not automatically allow the conclusion that ROS induces ATF4. The experiment I suggested in my previous report (i.e. determining whether ATF4 induction can be curbed by antioxidants) would have seemed easy enough to do to clearly demonstrate the validity of the model.
4. P 6, 2nd paragraph: The claim that "STAMP2 expression significantly increased PARP cleavage" is not supported by statistical testing. Number of replicates need to be stated and p value indicated.
5. P 5, 3rd paragraph refers to LAPC4 cells, although no experiments are shown with LAPC4.

Referee #2 (Comments on Novelty/Model System):

LNCaP, VCaP and LAPC4 act as relevant models of androgen-dependent and androgen-independent stages of prostate cancer.

Referee #2 (Remarks):

The authors have addressed several of the questions posed by the reviewers. However, two questions remain essentially unanswered.

Question # 1
Referee#2, 1. The scored immunohistochemical staining of STAMP2 in microarrayed human PCa tumors appears to correlate with the mRNA expression levels originally reported in their 2005 paper. However, the provided example of STAMP2 expression in benign prostate appears to have substantial expression in the luminal cells of the prostatic epithelium in what appears to be a membranous pattern (although the image quality provided to this reviewer was low), whereas all 27 of the benign samples were given a score of 0 in their membrane staining. No explanation of this discrepancy was provided.

Authors' response. A more representative picture for the benign prostate is now provided. The scoring was done such that no to very low expression was given a score of 0 which was the case in 23 out of 27 samples. This is now described more clearly in the Methods section, under 'Scoring and statistical analysis of TMAs'.

The authors have not addressed the specific question as to why in the original submission there was a sample of benign tissue that appeared to have membranous STAMP2 staining whereas the quantification indicated that all benign samples had a score of 0 for membranous staining. Does this relate to the IHC scoring system as described? The authors' own description of their scoring system indicates that "0 represents no staining by any tumor cells." [emphasis added] Benign tissue, by definition, would have no tumor cells. Thus, by this scoring system, the score for staining in benign tissue should not be anything other than 0. Is the score derived using a per-cell or per-field analysis of the specimen? If it is the latter, the score may be more a reflection of the percentage of tumor cells in the sample rather than differences in staining intensity within prostatic epithelial cells. If the entire field is used, a scoring system that is somehow normalized to the number of epithelial cells within an image rather than tumor cells seems appropriate. An epithelial cell specific marker (perhaps a pan-cytokeratin antibody?) should facilitate a more reasonable comparison across specimens.

Question # 2
Referee#2, 4. This reviewer questions whether the 3-5% reduction in relative ROS levels in response to decreased of STAMP2 expression in LNCaP cells (Fig. 6E) is sufficient to account for the biological effects being attributed to STAMP2 ferrireductase activity.

Authors' response. The reduction in ROS levels (measured by DHE staining), although limited in scope, is significant from experiments repeated several times. In the revised manuscript, we

confirmed this observation using a new oxidative stress detection reagent, CellROX® Deep Red Reagent (Life Technologies). As shown in Figure 9D, there was a significant increase (~3 fold) of ROS levels in LNCaP cells in response to androgen treatment. STAMP2 knockdown significantly decreased androgen induced ROS levels (10%), while there was no significant difference in the absence of androgen, i.e. when there was no/very low STAMP2 present. We have also obtained consistent results in 22RV1 cells (Supplementary Figure S5). Our interpretation of these robust data is that there may be a delicate balance of ROS in PCa cells which could be affected by relatively small changes induced by STAMP2. Another possibility is that the assays used have high basal level of detection sensitivity ('background') in these cell lines. Taken together, we feel that the data we present are robust and show that STAMP2 regulates ROS levels in PCa cells.

Referee#1, 5. The final model is not proven. The model predicts that androgen-mediated STAMP2-dependent ROS formation activates ATF4. It is shown that STAMP2 induces ROS and ATF4, but it remains unclear whether ATF4 induction is mediated by ROS. Do antioxidants inhibit ATF4 induction in response to STAMP2 overexpression (or indeed in response to androgen signaling)?

Authors' response. To show that ATF4 induction is mediated by ROS in PCa cells, we have used menadione which has been shown to increase ROS in other cell types (e.g. Perez Soler et al., Clin Cancer Res. 2011, v17: 6766-6777; Chowdury et al., Apoptosis. 2009, v14:108-123). As shown in Supplementary Figure S3, menadione treatment significantly increased ROS levels in LNCaP cells and induced ATF4 expression.

The dependence of STAMP2-induced ATF4 expression on increased ROS remains poorly supported by experimental evidence. Just because ROS can induce ATF4 expression doesn't mean that STAMP2 uses ROS to increase ATF4 expression. The authors appear to have the tools necessary to answer this question but have not yet done so. Fig 6C provides significant support for the importance of STAMP2 ferrireductase activity on LNCaP proliferation, assuming that equal expression levels of the WT and mutant were achieved. Does exogenous STAMP2 expression (WT or ferrireductase mutant dGSR) induce ATF4 expression in a ROS-dependent manner? I.e., does WT but not dGSR increase ATF4 expression in LNCaP? Does WT but not dGSR increase ROS in LNCaP cells? If these have been shown, are these effects generalizable to other cell types?

Referee #3 (Remarks):

The revised manuscript has successfully addressed all of my previous concerns. Namely, the inclusion of controls related to the hormone inducible expression and downstream effects of STAMP2 are more than satisfactory. Importantly, the authors have done an excellent job of expanding upon what was to me the most intriguing aspect of their original manuscript, that STAMP2 expression is restored upon the development of resistance to hormone therapy (human data) or castration (mouse data). This adds tremendous value to their story in my opinion. Reviewing others' comments, I find that the authors have also suitably addressed each concern raised in the original review. The additional data leaves very little unanswered. Perhaps the only missing information of interest to me is a data to suggest a mechanism of restored STAMP2 expression in CRPC. However, this is not compulsory, as the manuscript seems otherwise complete.

2nd Revision - authors' response 17 December 2014

Referee #1 (Remarks):

The authors have addressed a number of my previous comments and the manuscript is improved. Improvements include a documentation of the knockdown efficiency of endogenous STAMP2 protein, the inclusion of a mutant STAMP2 protein deficient in FAD binding, and the addition of size markers to most (not all) blots. The methods description was also improved, although the ferrireductase assay is still not described, and the statistical tests are insufficiently described.

The details of the ferrireductase assay are now described in Methods.

1. Fig. 4C and D is described as showing "statistically significant differences", although it was not indicated which statistical tests were done.

The statistical significance was determined by the t-test. The complete description is now placed in the figure legends for Fig. 4C and D.

2. Fig. 8H: It looks like there are no cells in the NBT negative dishes shown. Some sort of counterstaining should be shown to document the presence of cells in the negative dishes.

We have used crystal violet staining to show that there are similar number of cells in the plates (Supplementary Figure S4).

3. The final model in Fig. 10C remains unproven. The fact that STAMP2 induces ROS and that a chemical ROS inducer upregulates ATF4 does not automatically allow the conclusion that ROS induces ATF4. The experiment I suggested in my previous report (i.e. determining whether ATF4 induction can be curbed by antioxidants) would have seemed easy enough to do to clearly demonstrate the validity of the model.

We now provide data showing that ectopic expression of STAMP2 induced ATF4 expression (Supplementary Figure S3B) and this effect is inhibited by treating cells with the anti-oxidant N-acetyl cysteine in two different cell lines (Supplementary Figure S3B and S6C). Consistently, ectopic expression of a STAMP2 mutant, which has lost its ferrireductase activity as well as ROS generating ability, did not affect ATF4 expression (Supplementary Figure S6B). These data provide strong support for the model that we present in Figure 10C. We thank the reviewer for the suggestion which significantly strengthened our model.

4. P 6, 2nd paragraph: The claim that "STAMP2 expression significantly increased PARP cleavage" is not supported by statistical testing. Number of replicates need to be stated and p value indicated.

We have now reworded this sentence and also provided this information in Supplementary Figure S1A and 1B.

5. P 5, 3rd paragraph refers to LAPC4 cells, although no experiments are shown with LAPC4.

This section is now deleted. We apologize for this error.

Referee #2 (Comments on Novelty/Model System):

LNCaP, VCaP and LAPC4 act as relevant models of androgen-dependent and androgen-independent stages of prostate cancer.

Referee #2 (Remarks):

The authors have addressed several of the questions posed by the reviewers. However, two questions remain essentially unanswered.

Question # 1
Referee#2, 1. The scored immunohistochemical staining of STAMP2 in microarrayed human PCa tumors appears to correlate with the mRNA expression levels originally reported in their 2005 paper. However, the provided example of STAMP2 expression in benign prostate appears to have substantial expression in the luminal cells of the prostatic epithelium in what appears to be a membranous pattern (although the image quality provided to this reviewer was low), whereas all 27 of the benign samples were given a score of 0 in their membrane staining. No explanation of this discrepancy was provided.
Authors' response. A more representative picture for the benign prostate is now provided. The scoring was done such that no to very low expression was given a score of 0 which was the case in

23 out of 27 samples. This is now described more clearly in the Methods section, under 'Scoring and statistical analysis of TMAs'.
The authors have not addressed the specific question as to why in the original submission there was a sample of benign tissue that appeared to have membranous STAMP2 staining whereas the quantification indicated that all benign samples had a score of 0 for membranous staining. Does this relate to the IHC scoring system as described? The authors' own description of their scoring system indicates that "0 represents no staining by any tumor cells." [emphasis added] Benign tissue, by definition, would have no tumor cells. Thus, by this scoring system, the score for staining in benign tissue should not be anything other than 0. Is the score derived using a per-cell or per-field analysis of the specimen? If it is the latter, the score may be more a reflection of the percentage of tumor cells in the sample rather than differences in staining intensity within prostatic epithelial cells. If the entire field is used, a scoring system that is somehow normalized to the number of epithelial cells within an image rather than tumor cells seems appropriate. An epithelial cell specific marker (perhaps a pan-cytokeratin antibody?) should facilitate a more reasonable comparison across specimens.

In our scoring system, 0 referred to no apparent staining or very weak level of staining; this was corrected and pointed out in the revised manuscript (in Methods section, Scoring and statistical analysis of TMAs). Consistently, there is very low level of staining in the normal prostate sample that is presented in the figure. Since this scoring can, in retrospect, be misleading, that a sample with a 0 score can have very weak staining, in the revised manuscript we chose to change the score range from 1-4 instead of 0-3; there is no change to the data or their interpretation. We thank the reviewer for bringing this point up.

Question # 2
Referee#2, 4. This reviewer questions whether the 3-5% reduction in relative ROS levels in response to decreased of STAMP2 expression in LNCaP cells (Fig. 6E) is sufficient to account for the biological effects being attributed to STAMP2 ferrireductase activity.

Authors' response. The reduction in ROS levels (measured by DHE staining), although limited in scope, is significant from experiments repeated several times. In the revised manuscript, we confirmed this observation using a new oxidative stress detection reagent, called CellROX Deep Red Reagent (Life Technologies). As shown in Figure 9D, there was a significant increase (~3 fold) of ROS levels in LNCaP cells in response to androgen treatment. STAMP2 knockdown significantly decreased androgen induced ROS levels (10%), while there was no significant difference in the absence of androgen, i.e. when there was no/very low STAMP2 present. We have also obtained consistent results in 22RV1 cells (Supplementary Figure S5). Our interpretation of these robust data is that there may be a delicate balance of ROS in PCa cells which could be affected by relatively small changes induced by STAMP2. Another possibility is that the assays used have high basal level of detection sensitivity ('background') in these cell lines. Taken together, we feel that the data we present are robust and show that STAMP2 regulates ROS levels in PCa cells.

We would like to reiterate our response from before, that there is a significant decline in ROS levels in multiple experiments and in two different cell lines. These data suggest that STAMP2 is indeed involved in regulation of ROS levels in PCa cells. These data indicate that other pathways may also be involved in ROS regulation and the functional role of STAMP2 in PCa cells which needs to be further explored. This is now more clearly pointed out in the Discussion (p15, 2nd paragraph).

Referee#1, 5. The final model is not proven. The model predicts that androgen-mediated STAMP2-dependent ROS formation activates ATF4. It is shown that STAMP2 induces ROS and ATF4, but it remains unclear whether ATF4 induction is mediated by ROS. Do antioxidants inhibit ATF4 induction in response to STAMP2 overexpression (or indeed in response to androgen signaling)?

Authors' response. To show that ATF4 induction is mediated by ROS in PCa cells, we have used menadione which has been shown to increase ROS in other cell types (e.g. Perez Soler et al., Clin Cancer Res. 2011, v17: 6766-6777; Chowdury et al., Apoptosis. 2009, v14:108-123). As shown in Supplementary Figure S3, menadione treatment significantly increased ROS levels in LNCaP cells and induced ATF4 expression.

The dependence of STAMP2-induced ATF4 expression on increased ROS remains poorly supported

by experimental evidence. Just because ROS can induce ATF4 expression doesn't mean that STAMP2 uses ROS to increase ATF4 expression. The authors appear to have the tools necessary to answer this question but have not yet done so. Fig 6C provides significant support for the importance of STAMP2 ferrireductase activity on LNCaP proliferation, assuming that equal expression levels of the WT and mutant were achieved. Does exogenous STAMP2 expression (WT or ferrireductase mutant dGSR) induce ATF4 expression in a ROS-dependent manner? I.e., does WT but not dGSR increase ATF4 expression in LNCaP? Does WT but not dGSR increase ROS in LNCaP cells? If these have been shown, are these effects generalizable to other cell types?

As pointed out above (response to Reviewer 1, point 3), we have provided evidence that ectopic STAMP2 expression increased ATF4 expression (Supplementary Figure S3B) which is inhibited by the anti-oxidant N-acetyl cysteine (NAC) (Supplementary Figure S3B and S6C-D). Consistently, ectopic expression of a STAMP2 mutant, with no ferrireductase activity or ROS generating ability, did not affect ATF4 expression (Supplementary Figure S6E). In addition, NAC significantly blocked ROS generation by STAMP2 in the NBT reduction assay (Supplementary Figure S5). Regarding the ROS generation capability of wild type STAMP2 compared with the dGSR mutant, these data were already provided in the previous version of the manuscript (Figure 9C).

Referee #3 (Remarks):

The revised manuscript has successfully addressed all of my previous concerns. Namely, the inclusion of controls related to the hormone inducible expression and downstream effects of STAMP2 are more than satisfactory. Importantly, the authors have done an excellent job of expanding upon what was to me the most intriguing aspect of their original manuscript, that STAMP2 expression is restored upon the development of resistance to hormone therapy (human data) or castration (mouse data). This adds tremendous value to their story in my opinion. Reviewing others' comments, I find that the authors have also suitably addressed each concern raised in the original review. The additional data leaves very little unanswered. Perhaps the only missing information of interest to me is a data to suggest a mechanism of restored STAMP2 expression in CRPC. However, this is not compulsory, as the manuscript seems otherwise complete.

We thank the reviewer for finding our manuscript suitable for publication in EMBO Mol Med. We are very much interested in the mechanism of restored STAMP2 expression in CRPC, but currently do not have any firm data on this.

We thank the reviewers for their comments which significantly improved our manuscript. We hope that you and the reviewers will now find our manuscript acceptable for publication in EMBO Molecular Medicine.

3rd Editorial Decision 08 January 2015

Thank you for the submission of your revised manuscript to EMBO Molecular Medicine. We have now received the enclosed reports from the referees that were asked to re-assess it. As you will see the reviewers are now globally supportive and I am pleased to inform you that we will be able to accept your manuscript pending the following final amendments:

1) Please address Reviewer 2's remaining concerns on statistical issues. Connected to this, and as per our Author Guidelines, the description of all reported data that includes statistical testing must state the name of the statistical test used to generate error bars and P values, the number (n) of independent experiments underlying each data point (not replicate measures of one sample), and the actual P value for each test (not merely 'significant' or 'P < 0.05').

Please submit your revised manuscript within two weeks. I look forward to seeing a revised form of your manuscript as soon as possible.

I look forward to reading a new revised version of your manuscript as soon as possible.

Referee #1 (Remarks):

The new studies have substantially strengthened the model, especially the demonstration that overexpression of STMAP2 induces ATF4 and that this effect is inhibited by antioxidants.

Referee #2 (Remarks):

The model proposed by the authors, that androgen-regulated STAMP2 expression in prostate cancer leads to enhanced proliferation and survival through its ferroxireductase activity, is novel and interesting. The authors do a good job of providing evidence to support this hypothesis, showing that the loss of STAMP2 affects survival and proliferation of androgen-sensitive LNCaP and VCaP cells in vitro and in vivo and that STAMP2 possesses ferroxireductase activity. The correlation of STAMP2 and ATF4 in various prostate cancer specimens and model cell lines under various conditions further supports this hypothesis. The addition to the most recent version of the manuscript of Figures S3 and S6 significantly strengthens the authors' conclusions; the authors demonstrate that ATF4 expression is increased in response to expression of STAMP2 but not the ferroxireductase-deficient mutant and this effect is inhibited by N-acetylcysteine (presumably by increasing intracellular antioxidant glutathione levels), supporting the hypothesis that the ferroxireductase activity of STAMP2 promotes PCa progression by increasing ATF4 levels. Overall, the manuscript is sufficiently improved over the initial submission to be acceptable for publication but would still benefit from a review of the statistical considerations by a true statistician (although most of the data shown are sufficiently strong that more appropriate statistical testing would maintain consistency with the authors' interpretations.) The authors have provided ample support that STAMP2, ATF4 and reactive oxygen species (in general) contribute to the progression of prostate cancer and warrant further investigation.

Minor points

Fig. 1B. The t-test is inappropriate statistical test for comparing multiple independent variables; one-way anova with a post hoc test would be more suitable. In addition, the y-axis should be updated to reflect the new scale (1-4 rather than 0-3). Same for Fig. 4B.

page 11, first paragraph: N-acetyl cysteine is misspelled.

Fig. 4D. T-test is inappropriate statistical test for comparing multiple independent variables; one-way anova with a post hoc test appears more suitable.

When reporting P values, the authors should indicate the statistical tests performed from which P values are obtained (e.g. in the figure legends).

3rd Revision - authors' response 14 January 2015

Response to reviewer´s comments:

#1. Fig. 1B. The t-test is inappropriate statistical test for comparing multiple independent variables; one-way anova with a post hoc test would be more suitable. In addition, the y-axis should be updated to reflect the new scale (1-4 rather than 0-3). Same for Fig. 4B.

We have now confirmed the statistical significance of the data presented in Fig. 1B by one-way Anova as requested by the reviewer which is described in the legend of Figure 1B. In addition, the y-axis of Figures 1B and 4B are updated according to the new scale. We thank the reviewer for pointing these out.

#2. page 11, first paragraph: N-acetyl cysteine is misspelled.

The error has now been corrected.

#3 .Fig. 4D. T-test is inappropriate statistical test for comparing multiple independent variables; one-way anova with a post hoc test appears more suitable.

As suggested by the reviewer, we have now used one-way Anova to calculate the significance as described in the legend of Figure 4B.

#4. When reporting P values, the authors should indicate the statistical tests performed from which P values are obtained (e.g. in the figure legends).

The information on the statistical tests used and the exact P values are now provided in the figure legends. In some cases, when the significance is very high, the statistical analysis program we used only resulted in $P>0.0001$ which was then noted.
